# The Anisotropic Complex Dielectric Function of CsPbBr_3_ Perovskite Nanorods Obtained via an Iterative Matrix Inversion Method

**DOI:** 10.1021/acs.jpcc.3c03423

**Published:** 2023-07-21

**Authors:** Freddy A. Rodríguez Ortiz, Boqin Zhao, Je-Ruei Wen, Ju Eun Yim, Giselle Bauer, Anna Champ, Matthew T. Sheldon

**Affiliations:** † Department of Chemistry, Texas A&M University, College Station, Texas 77843, United States; ‡ Department of Materials Science and Engineering, Texas A&M University, College Station, Texas 77843, United States

## Abstract

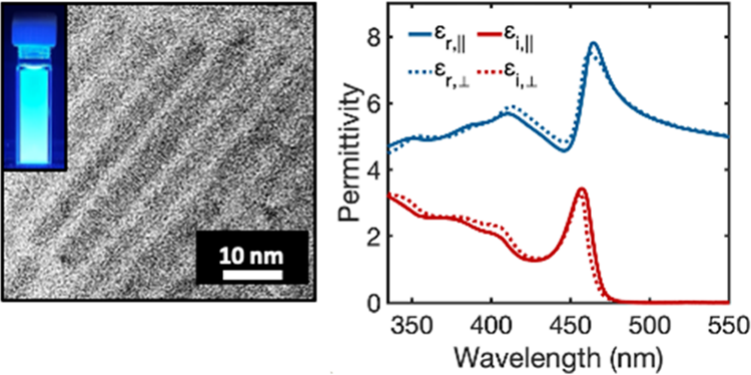

Colloidal lead halide perovskite nanorods have recently emerged as promising optoelectronic materials. However, more information about how shape anisotropy impacts their complex dielectric function is required to aid the development of applications that take advantage of the strongly polarized absorption and emission. Here, we have determined the anisotropy of the complex dielectric function of CsPbBr_3_ nanorods by analyzing the ensemble absorption spectra in conjunction with the ensemble spectral fluorescence anisotropy. This strategy allows us to distinguish the absorption of light parallel and perpendicular to the main axis so that the real and imaginary components of the dielectric function along each direction can be determined by the use of an iterative matrix inversion (IMI) methodology. We find that quantum confinement gives rise to unique axis-dependent electronic features in the dielectric function that increase the overall fluorescence anisotropy in addition to the optical anisotropy that results from particle shape, even in the absence of quantum confinement. Further, the procedure outlined here provides a strategy for obtaining anisotropic complex dielectric functions of colloidal materials of varying composition and aspect ratios using ensemble solution-phase spectroscopy.

## Introduction

Colloidal lead halide perovskite (LHP) nanocrystals (NCs) with the composition CsPbX_3_ (X = Cl, Br, or I) are a promising class of fluorescent emitters for diverse optoelectronic applications.^1−4^ These materials exhibit high luminescence quantum yields and narrow emission bandwidths that can be tuned across the visible spectrum through size-dependent quantum confinement and composition.^5−7^ Furthermore, recent advances in their colloidal synthesis have enabled the fabrication of anisotropic morphologies, such as nanowires,^8−12^ nanorods,^13−15^ and nanoplatelets,^16,17^ resulting in structures with specifically tailored optoelectronic functionalities.

Among these structures, LHP nanorods (NRs) with a one-dimensional (1D) morphology are of particular interest. Due to their restricted diameter, photogenerated excitons exhibit strong spatial confinement in two dimensions, with more electronic delocalization along the long axis of the nanorod. Compared to isotropic-shaped LHP nanocrystals, NRs show distinct attributes such as larger absorption cross sections,^18^ reduced amplified spontaneous emission,^19,20^ improved charge transport,^21,22^ and other more ideal optoelectronic properties. Recent reports have also demonstrated that LHP nanorods display strong optical anisotropy in the form of linearly polarized absorption and emission of light,^23−25^ making them attractive for applications in liquid-crystal devices (LCDs)^26−28^ and luminescent solar concentrators (LSCs).^29−32^ To continue the rapid progress in developing optoelectronic and photonic devices containing LHP NRs, detailed knowledge of the complex dielectric function is crucial for predicting the performance and forming strategies for device architecture optimization. However, despite the outstanding optoelectronic properties identified in experiments, the complex dielectric function of LHP NRs remains unknown.

To date, the majority of studies that have determined the complex dielectric function of LHP materials have focused on bulk single crystals^33−35^ and thin films.^36,37^ Moreover, those studies are based on spectroscopic ellipsometry, which requires flat, optically smooth surfaces and uniform samples deposited on a standard substrate. In addition, accurate interpretation of ellipsometry data typically requires the use of complex optical models to describe the optical properties of substrates, intermediate layers, and surface roughness, which can be challenging and time-consuming.^38^ Thus, spectroscopic ellipsometry is not an appropriate characterization method for obtaining the complex dielectric function of colloidal nanocrystals.

Previous studies have proposed alternative methodologies for extracting the complex dielectric function of colloidal nanocrystals based on their collective absorption spectrum.^39,40^ In particular, Moreels et al. developed a method for extracting the complex dielectric function of colloidal nanocrystals using the ensemble, solution-phase absorption spectrum.^40^ Their method involves an iterative minimization procedure that combines Maxwell–Garnett (MG) effective medium theory with Kramers–Kronig (KK) relations to obtain both the real and imaginary components of the dielectric functions. The main idea of this method is to systematically improve a trial dielectric function until the theoretical absorption coefficient spectrum of the nanocrystal is accurately reproduced in the experimental range. Thus far, this approach has been applied to determine the complex dielectric function of isotropic-shaped nanoparticles, including spherical-shaped lead chalcogenides nanoparticles,^40,41^ and, more recently, cuboidal-shaped LHP nanocrystals.^42^ For isotropic-shaped nanoparticles, the complex dielectric function is similar for all polarization directions, and thus light absorption by the nanoparticle is isotropic. Because of the isotropic absorption, initial studies used the solution-state absorption spectrum of randomly oriented ensembles of isotropically shaped nanoparticles to determine the complex dielectric function. However, the use of a modified version of this procedure for determining the complex dielectric function of the nanorods has not yet been reported.

For 1D nanorods, the complex dielectric function is anisotropic, owing to the distinct density of states for directions parallel and perpendicular to the main nanorod axis. This anisotropy of density of states is particularly pronounced for energies near the band edge, where quantum confinement effects strongly modulate the electronic band structure.^43−46^ Because the ensemble absorption spectrum of randomly dispersed nanorods is a weighted average over the parallel and perpendicular directions to the nanorod main axis, calculation of the complex dielectric function from the ensemble absorption spectrum yields the so-called isotropic dielectric function (i.e., average dielectric function over all nanorod orientations). In order to determine the anisotropic dielectric function of nanorods through an iterative procedure, information about the distinct absorption spectra along the long and short axes of the nanorod must be known. In theory, the absorption spectra along each axis can be measured through single-particle absorption experiments. However, direct determination of the absorption spectra of single nanoparticles is inherently difficult, given their small absorption cross section. An alternative method for obtaining information about the anisotropic absorption properties is fluorescence anisotropy spectroscopy. In rod-shaped nanoparticles, the optical properties are dictated by the ellipsoidal/cylindrical symmetry, resulting in optical transitions with polarization parallel and perpendicular to the nanorod main axis.^47−50^ Thus, from fluorescence anisotropy measurements, determination of the absorption anisotropy and information about light absorption along each axis of the nanorod can be obtained.

In this study, we present a strategy to determine the anisotropic complex dielectric function of the CsPbBr_3_ nanorods. Our approach uses the ensemble fluorescence anisotropy spectrum in combination with the ensemble absorption spectrum, in order to obtain information about the absorption spectra along the long and short axes of the nanorod. With these spectra we determine the anisotropic complex dielectric function using an iterative procedure adapted from Moreels et al.^40^ The resultant anisotropic dielectric function shows how the effects from quantum confinement of the electronic structure and, separately, 1D shape anisotropy contribute to the optical properties of CsPbBr_3_ nanorods.

## Experimental Section

### Materials

Cesium carbonate (Cs_2_CO_3_, 99.995% trace-metal basis), lead oxide (PbO, 99.999% trace-metal basis), lead(II) bromide (PbBr_2_, 98%), oleic acid (OA, technical grade 90%), oleylamine (OAm, technical grade 70%), 1-octadecene (ODE, technical grade 90%), toluene (anhydrous, 99.8%), and hexane (95%) were received from Sigma-Aldrich. Hydrobromic acid (HBr, 48%) was purchased from VWR Chemicals BDH. OA and OAm were dried with molecular sieves under an argon atmosphere before use. Other chemicals were used as received.

### Synthesis of CsPbBr_3_ Nanorods via Slow Injection of Precursors

For this study, cesium lead bromide nanorods were prepared by employing a dual slow injection technique previously reported by our group.^13^ Briefly, the ODE (5 mL) was added to a 25 mL round-bottom flask and dried under vacuum at 120 °C. After 1 h, the ODE was allowed to cool to 80 °C and 0.3 mL of a Pb-OA stock solution was swiftly injected into the flask. Next, a Cs precursor solution was prepared by mixing 0.3 mL of the Cs-OA stock solution with OA (0.55 mL), OAm (0.25 mL), and ODE (0.27 mL). Separately, a Br precursor solution was made by dispersing 0.085 g of OAm-Br (0.085 g) with toluene (0.65 mL) and ODE (0.65 mL). The two precursor solutions, with a total volume of 0.9 mL each, were then separately injected into the reaction flask at an injection rate of 10.8 mL/h. After the reaction was completed, the product was purified by centrifugation at 8000*g*-forces for 15 min. The supernatant was discarded, and the precipitate was then redispersed in hexanes followed by centrifugation at 3000*g*-forces for another 10 min. The final supernatant was collected for further analysis.

### Synthesis of CsPbBr_3_ Nanocubes

Cube-shaped cesium lead bromide nanocrystals were prepared by modifying a previously published hot-injection method.^4^ Briefly, Cs_2_CO_3_ (0.200 g), OA (0.624 mL), and ODE (10 mL) were added to a 25 mL three-neck round-bottom flask and heated for 1 h at 120 °C under vacuum. After 1 h, the flask was put under argon and heated to 150 °C until all of the Cs_2_CO_3_ had reacted to form Cs-oleate. The lead bromide precursor solution was prepared by mixing 0.376 mmol of PbBr_2_ (0.138 g) with ODE (10 mL) in a 25 mL three-neck round-bottom flask and heated under vacuum to 120 °C for 1 h. The solution was then placed under argon, and dried OA (1.0 mL) and OA (1.0 mL) were injected to solubilize the PbBr_2_ salt. The temperature was then increased to 175 °C, and the Cs-oleate (0.8 mL) was swiftly injected. After 5 s, the solution was cooled with an ice bath, the final crude solution was centrifuged at 3000*g*-forces for 10 min, and the supernatant was discarded. The precipitate was then cleaned three times using a combination of ODE and hexane. The precipitates were suspended in 2 mL of anhydrous hexane for further analysis.

### Characterization

Powder X-ray diffraction (XRD) measurements were performed using a BRUKER D8-Focus Bragg–Brentano X-ray powder diffractometer equipped with a Cu Kα radiation source (λ = 1.5418 Å). Absorption and photoluminescence spectra were collected on an Ocean Optics Flame-S-UV–vis Spectrometer with an Ocean Optics DH-200-Bal deuterium and halogen lamp as the light source. High-resolution transmission electron microscopy (HRTEM) images were taken on an FEI Tecnai G2 F20 ST FE-TEM operated at 200 kV equipped with a Gatan CCD camera.

### Optical Measurements of Ensembles

Fluorescence anisotropy, photoluminescence excitation spectroscopy, and fluorescent lifetime measurements were performed by using a Photon Technology International (PTI) QuantaMaster 40 spectrofluorometer. Fluorescence anisotropy of ensemble samples was measured by using an L-format fluorometer configuration equipped with a steady-state xenon arc lamp excitation, computer-controlled Glan-Thompson polarizers, and steady-state photomultiplier tube (PMT) detection system. Photoluminescence excitation spectra were measured by exciting ensemble samples across the visible and recording emission spectra. Fluorescent lifetime measurements were performed using a time-domain stroboscopic detection system equipped with a 435 nm LED pulsed laser. All fluorescence anisotropy measurements were performed using diluted solutions to avoid multiple scattering and/or multiple reabsorption and reemission events that can decrease the measured fluorescence anisotropy.

## Results and Discussion

### Characterization of CsPbBr_3_ Nanorods

**Figure 1:**
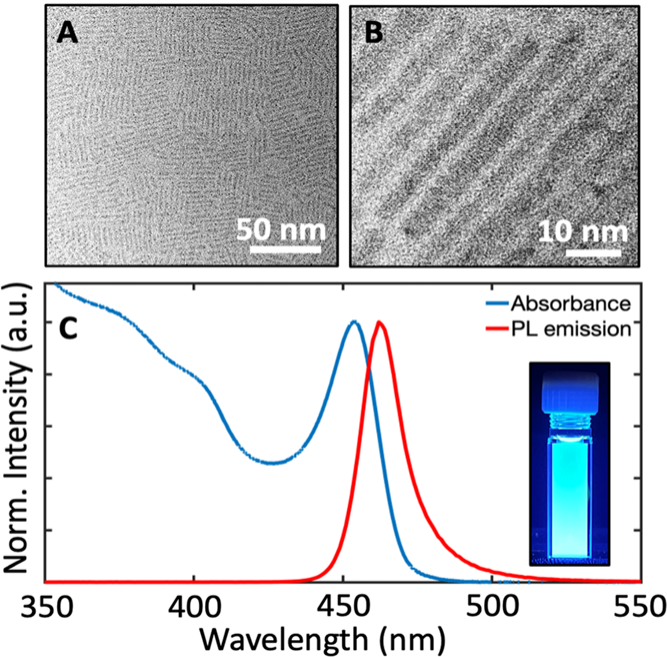
(A, B) Transmission electron micrographs and (C) absorption and photoluminescence emission spectra of CsPbBr_3_ nanorods (inset: photograph of a solution of nanorods under ultraviolet illumination).

Ensembles of CsPbBr_3_ nanorods were synthesized using a recently developed method that leverages the slow, simultaneous injection of precursors to precisely control the size and aspect ratio across the quantum confinement regime.^13^Figure 1A–C highlights the morphological and optical properties of CsPbBr_3_ nanorods. These materials exhibit highly uniform dimensions, with an average length of 40.2 ± 5.1 nm and width of 3.91 ± 0.56 nm, as determined by transmission electron microscopy (see Figures S1 and S2 for additional TEM images and size distribution analysis). XRD diffraction measurements indicate that these materials adopt the orthorhombic (*Pbnm*) CsPbBr_3_ phase structure, consistent with our previous results on the same material (see Figure S3).^13^ The absorption spectrum in Figure 1C displays a strong and well-resolved excitonic peak, indicating strong confinement of excitonic carriers and high ensemble uniformity. Interestingly, the photoluminescence (PL) spectrum shows a narrow line width emission peak (full-width half-maximum ∼85 meV) with asymmetric broadening on the low energy side. These asymmetric PL spectra have been observed in strongly confined 1D and 2D CsPbBr_3_ nanocrystals and have been attributed to several mechanisms, including size polydispersity and emission from localized states.^16,51−53^ In this study, the observed asymmetric PL broadening is likely due to localized state emission, based on the high sample uniformity observed by TEM, the sharp absorption edge, and photoluminescence excitation (PLE) and PL lifetime studies (see Figures S4 and S5 and discussion in the Supporting Information).

### Ensemble Fluorescence Anisotropy of CsPbBr_3_ Nanorods

**Figure 2:**
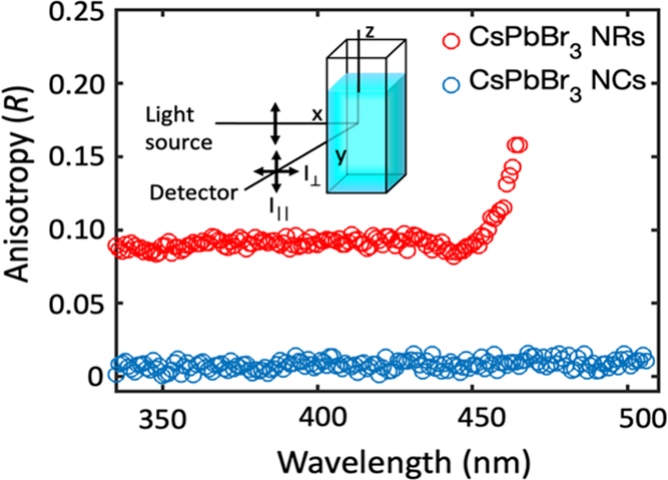
Ensemble fluorescence anisotropy spectra acquired for CsPbBr_3_ nanorods (red circles) and CsPbBr_3_ nanocubes (blue circles). The inset depicts a schematic diagram for measurements of fluorescence anisotropy using the photoselection method.

To study the polarized optical properties of CsPbBr_3_ nanorods, we follow the work of Sitt et al. and others^47−49^ who analyzed colloidal nanorods using a photoselection method (inset in Figure 2). In this technique, a random ensemble of particles is illuminated with vertically polarized light, which selectively excites a population of particles with absorption transition dipoles that are parallel to the electric vector of excitation. The selective excitation results in a partially oriented population of excited particles (i.e., photoselection) parallel to the electric vector of excitation. The photoluminescence intensity from the excited particles is monitored perpendicular to the excitation polarization direction through vertical and horizontal optical polarizers. The ensemble fluorescence anisotropy (*R*) at a given wavelength is calculated from the ratio of the intensities according to eq 1
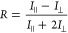
1where *I*_||_ and *I*_⊥_ are the vertically and horizontally polarized emission intensities, respectively.

Fluorescence anisotropy describes the relationship between absorption and emission polarizations. For a random ensemble of particles, possible anisotropy values can range from 0.4 if both the absorption and emission are fully linearly polarized in the same direction to −0.2 if the absorption and emission are completely polarized in perpendicular directions. Samples that do not have defined absorption and emission polarizations show values of *R* = 0. By the photoselection method, the excitation energy is varied while the emission is measured at a specific energy. Thus, from photoselection measurements, information about the polarization relationship of higher-energy absorption transitions compared with the band-edge emission can be derived. In particular, each maximum in the spectrum corresponds to an optical transition that is polarized parallel to the emission, while each minimum corresponds to an optical transition that is polarized perpendicular to the emission.

Using the photoselection technique, we measure the fluorescence anisotropy spectra by exciting samples across the ultraviolet and visible and recording the emission intensities at the emission peak maximum according to eq 1. Figure 2 shows the fluorescence anisotropy spectrum acquired for CsPbBr_3_ nanorods (red open dots). For comparison, the fluorescence anisotropy of conventional CsPbBr_3_ nanocubes with an average edge size of 8.5 nm was also measured (blue open dots; see Figures S6 and S8 for TEM images, size distribution histogram, and XRD diffractogram). Interestingly, the fluorescence anisotropy spectrum of nanorods exhibits positive, variable anisotropy values with the highest anisotropy (*R* ∼ 0.17) for near-band-edge excitation, which decreases rapidly and recovers to a constant value (*R* ∼ 0.09) at high excitation energies. In contrast, CsPbBr_3_ nanocubes show a featureless fluorescence anisotropy spectrum with constant values at *R* = 0, indicating no anisotropy, as previously observed.^24^ We note that the fluorescence lifetime of CsPbBr_3_ nanorods (18 ns) is significantly faster than the rotational diffusion rates of nanorods (greater than 1 μs), and thus, depolarization of anisotropy due to Brownian motions can be regarded as negligible (see Figure S9 and discussion in the Supporting Information).

The measured anisotropy of CsPbBr_3_ nanorods was found to differ significantly from other related reports, particularly for excitations near the band edge. For instance, a recent study by Dou et al. reported relatively constant (*R* = 0.1) and featureless fluorescence anisotropy spectra for ensemble solutions of CsPbBr_3_ nanorods with an average length of 32 nm and width of 8.3 nm.^23^ Nanorods in this study, however, display a fluorescence anisotropy pattern similar to that observed for quantum-confined CdSe nanorods: the highest anisotropy is at the lowest-energy excitation, followed by a dip and constant lower values at high excitation energies.^47,48,54^ In CdSe nanorods, the large band-edge anisotropy has been attributed to the strong polarization of quantized transition dipoles of the lowest-excited state along the nanorod’s long axis. The similarity of the anisotropy pattern compared to quantum-confined CdSe nanorods is indicative of the role of quantum confinement on the band-edge polarization properties of CsPbBr_3_ nanorods. Indeed, recent reports on the electronic structure of 1D nanowires and slightly elongated cuboids showing quantum confinement effects have demonstrated that the lowest-excited exciton state displays strong polarization along the main axis of the nanocrystal.^55,56^

As mentioned earlier, in rod-shaped nanocrystals, the polarization components of absorption and emission are determined by the ellipsoidal symmetry, resulting in a *z*-component along the nanorod and equal *x* and *y* components that exhibit planar polarization perpendicular to the nanorod. Hence, the ensemble fluorescence anisotropy of isotopically oriented nanorods measured using photoselection is a function of both the anisotropy of absorption (*r* = (*r*_||_ – *r*_⊥_)/(*r*_||_ + 2*r*_⊥_)) and anisotropy of emission (*q* = (*q*_||_ – *q*_⊥_)/(*q*_||_ + 2*q*_⊥_)) of single nanorod,^47^ according to
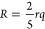
2

Here, the 2/5 prefactor describes the loss of anisotropy due to the photoselection of a randomly oriented ensemble, and *r* and *q* represent the absorption and emission anisotropies, respectively (Supporting Information for a detailed description and full derivation of eq 2). Values of *r* or *q* can range from 1, for fully polarized intensities along the main axis (*z* axis) to −0.5, for equal intensities along the minor axes (xy plane). Because nanorod emission derives from the same band-edge states regardless of the excitation energy, the anisotropy of the emission (*q*) is fixed, while the absorption anisotropy (*r*) and *R* depend on the energy of excitation. Direct measurement and/or plausible assumptions of any of two *R*, *r*, or *q* allow calculation of the third by eq 2.

In colloidal nanorods, absorption anisotropy is determined by the interplay between (1) anisotropic transition dipoles of the band structure and (2) classical dielectric confinement effects. Anisotropic transition dipoles are associated with the absorption by electronic transitions with polarization parallel and perpendicular to the nanorod long axis and are directly related to the complex dielectric function (i.e., joint density of states) along the two axes. In contrast, dielectric effects result from the anisotropic confinement of the optical electric field inside the nanorod material due to differences in dielectric constant between the nanorod and the surrounding environment.^47,57,58^ When a nanorod with a high dielectric constant is placed in an external environment with a low dielectric constant, the electric field inside the nanorod is significantly reduced for incident electric fields polarized perpendicular to the nanorod’s main axis, while it is hardly affected for polarizations parallel to the long axis. This anisotropic distribution of the electric field leads to the preferential alignment of transition dipoles along the long axis, resulting in absorption anisotropy. The contribution of these effects to the absorption intensities along each axis of the nanorod can be described by using Maxwell–Garnett effective medium theory (see below). Because these effects are directly related to the complex dielectric function of the nanorod, information on absorption anisotropy can be used to determine the anisotropic complex dielectric function by modifying an iterative method, as described below.

### Calculation of the Full Complex Dielectric Function of CsPbBr_3_ Nanorods

**Figure 3:**
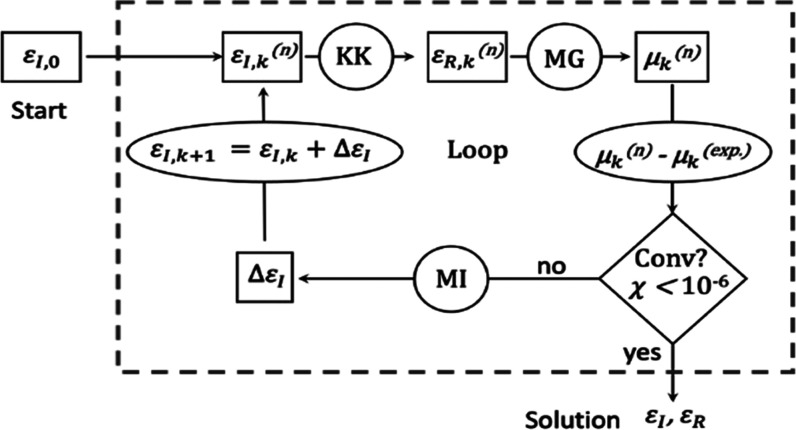
Schematic diagram of the iterative matrix inversion method. KK: Kramers–Kronig transformation; MG: calculation of μ according to Maxwell–Garnett effective medium theory; MI: matrix inversion.

As mentioned above, the complex dielectric function of nanocrystals can be determined from the experimental absorption spectrum by implementing an iterative matrix inversion method (IMI) that combines Maxwell–Garnett (MG) effective medium theory with the analysis of Kramers–Kronig relations (Figure 3). A detailed description of the IMI method can be found in the report of Moreels et al.^40^ Here, we extend this methodology to obtain the complex dielectric function of rod-shaped CsPbBr_3_ nanocrystals. For the sake of brevity, derivation of expressions used in the iterative method for the calculation of the dielectric function is provided in the Supporting Information.

In the conventional IMI method, the dielectric function of the nanocrystals is obtained from the absorption spectrum of a randomly distributed ensemble of nanocrystals. We first consider the calculation of the isotropic complex dielectric function of randomly oriented nanorods from the ensemble absorption spectrum and then describe the use of the optical anisotropy spectrum to determine the anisotropic complex dielectric function.

In a colloidal nanocrystal dispersion, the electric field within the nanocrystal will change based on the refractive index of its surroundings. For nanocrystal systems, the nanocrystal will generally have a higher refractive index than those of the surroundings. This leads to dielectric screening and a reduction in the local electric field inside the nanocrystal relative to the external electric field. The degree of dielectric screening is dependent on the shape of the nanocrystal. For 1D nanorods modeled as prolate ellipsoids of rotation, external electric fields along the short axis are significantly screened, while minimal screening occurs along the main axis, as mentioned above. The relationship between the absorption coefficient and the complex dielectric function, accounting for dielectric screening effects, can be expressed by using the MG mixing rule. The absorption coefficient (μ_*i*,NR(ave.)_) of randomly dispersed nanorod inclusions over the wavelength of interest according to the MG mixing rule can be defined as
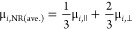
3where
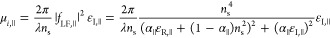
4
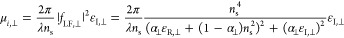
5

Here, *f*_LF,||_ and *f*_LF,⊥_ denote the parallel and perpendicular local field factors, respectively; *n*_s_ is the refractive index of the solvent (in our case this is hexane, *n*_s_ = 1.3749); α_||_ and α_⊥_ are the parallel and perpendicular depolarization factors, respectively; and ε_R,||_, ε_R,⊥_ and ε_I,||_, ε_I,⊥_ are real and imaginary parts of the dielectric function for directions parallel and perpendicular to the nanorod long axis, respectively. In our calculation, we modeled CsPbBr_3_ nanorods as prolate ellipsoids with a major axis of 40 nm and a minor axis of 4 nm based on size analysis of electron micrographs (Figures 1 and S2). For this aspect ratio, the depolarization factors were calculated to be α_||_ = 0.0202 and α_⊥_ = 0.4898 (more information can be found in the Supporting Information). However, it is important to note that the IMI procedure provides a dielectric function that reflects the statistical distribution of particle sizes contained in the sample.

**Figure 4:**
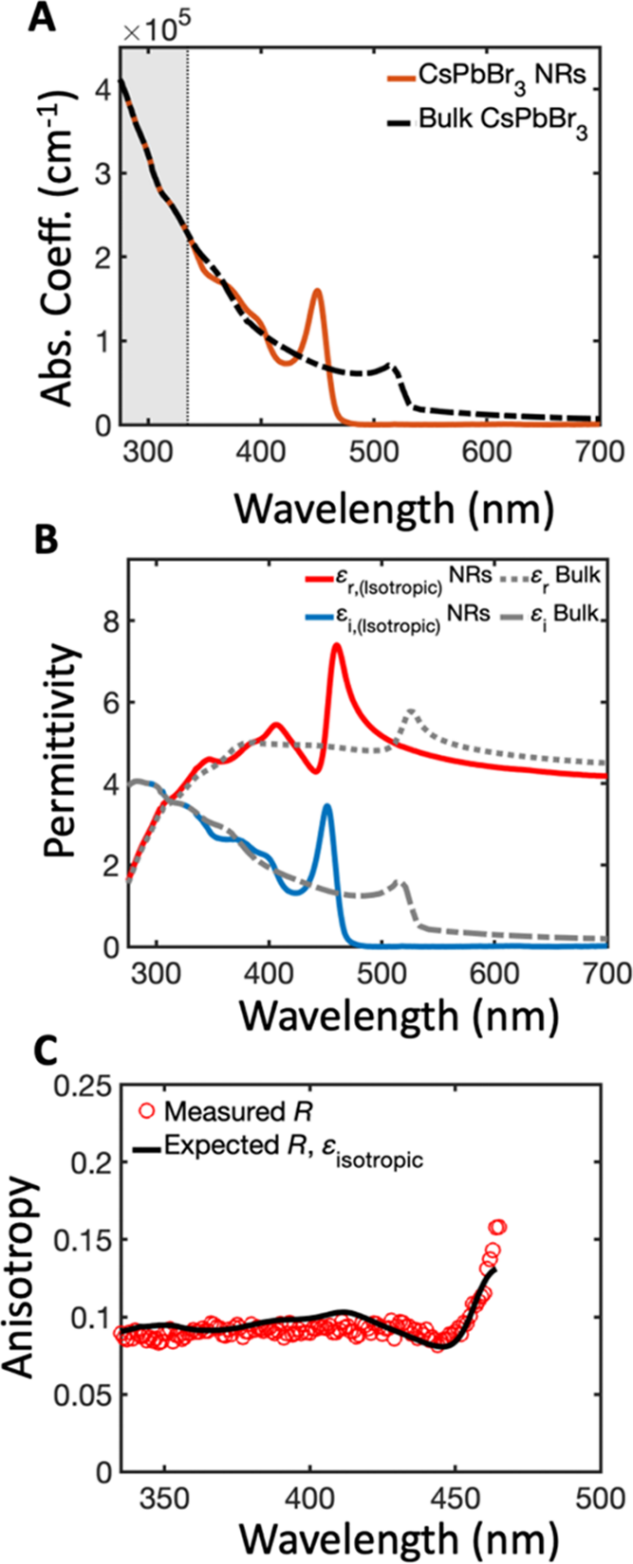
(A) Average absorption coefficient as a function of wavelength for CsPbBr_3_ nanorods in hexane as determined by Maxwell–Garnett effective medium theory (orange solid curve). The absorption coefficient is normalized at 335 nm to the bulk value (black dashed curve). (B) Isotropic real (red trace) and imaginary (blue trace) dielectric portions of CsPbBr_3_ nanorod found after performing the IMI method. The isotropic dielectric functions of CsPbBr_3_ nanorod are compared to the bulk dielectric functions (dotted and dash-dotted gray trace). (C) Expected ensemble anisotropy (*R*-expected) calculated from the absorption anisotropy using the isotropic dielectric function.

First, in order to determine the complex dielectric function of CsPbBr_3_ nanorods, we need its absorption coefficient (μ*_i_*) spectrum. Previous studies have demonstrated that the absorption coefficient of nanocrystals approaches bulk values at energies well above the band edge.^58−61^ As a result, the μ*_i_* spectrum can be obtained by normalizing the experimental absorption spectrum to bulk values at a reference energy. We started by measuring the absorption spectrum of a dilute solution of CsPbBr_3_ nanorods dispersed in hexane. Consistent with previous studies, we note that at short wavelengths (335 nm and below), features in the absorption spectrum of nanorods closely resemble bulk-like inclusions of CsPbBr_3_ in solution (i.e., μ spectrum determined by eq 3 using the refractive index of hexane, calculated depolarization factors, and experimental dielectric function of bulk CsPbBr_3_).^34^ Therefore, we obtained μ*_i_* of nanorods by normalizing the experimental absorption spectrum at 335 nm to its bulk μ value (μ_bulk_ = 2.28 × 10^5^ cm^–1^) (Figure 4A).

The μ*_i_* spectrum now allows the calculation of the real and imaginary parts of the dielectric function using the IMI method. For our initial trial function (ε_I,0_), we used the experimental bulk dielectric values of CsPbBr_3_ determined from spectroscopic ellipsometry by Mannino et al.^34^ As mentioned above, at short wavelengths (335 nm and below), transitions are essentially bulk-like, suggesting that ε values for the nanorods are identical to bulk ε values. Therefore, we assume that ε_I,NR_ is the same as ε_I,bulk_ below 335 nm (see more information in the Supporting Information). Moreover, we set the upper wavelength limit to 800 nm for the IMI calculation, as at this wavelength, CsPbBr3 nanorods do not absorb light, and therefore, ε_I_ does not contribute to ε_R_ (ε_I_ = 0).

From the initial ε_I,0_, the real part of the dielectric function is calculated by KK relations. We used the discrete form of the KK relations to transform new trial functions of ε_I_ into the corresponding KK ε_R_ of the dielectric function, thereby ensuring that the calculated permittivity values obey the KK relations. This initial set of dielectric functions is then used to calculate the theoretical absorption coefficient (μ*_k_*) values according to MG effective medium theory (eq 3). The theoretical μ_*k*_ values are then compared to the experimental absorption coefficient (μ_*k*,exp._, Figure 4A) by calculating the root-mean-square error (RMSE, χ). The complex dielectric function is obtained when the χ value between μ*_k_* and μ_*k*(exp.)_ is less than 10^–6^. If the difference between μ*_k_* and μ*_k_*_(exp.)_ is not less than 10^–6^, new trial imaginary dielectric functions (ε_I*,k*+1_ = ε_I*,k*_ + Δε_I_) are produced by matrix inversion (more information can be found in SI). A new set of dielectric functions are then calculated using KK relations and used in the next iterative step. This process is repeated until the difference in χ between μ*_k_* and μ*_k_*_(exp.)_ in successive iterations is reduced to values below 10^–6^.

Figure 4B shows the resulting isotropic real and imaginary parts of the dielectric function for randomly oriented CsPbBr_3_ nanorods after 3 iterative steps (steps are illustrated in Figure S12) along with values of bulk CsPbBr_3_. We observe sharp features in both the real and imaginary parts of the dielectric functions that are directly related to peaks in the absorption spectra. These spectral features contrast with the much smoother components of the bulk dielectric function, indicative of the discrete density of states induced by quantum confinement. Additionally, we observe a blue shift for the lowest-energy transition of ε_I_ that is accompanied by an increase in the oscillator strength with respect to the bulk.

Using this calculated, isotropic dielectric function, ε_isotropic_, we modeled the expected ensemble anisotropy, *R*-expected, according to the MG effective medium theory and eq 2, and compared it to the experimentally measured anisotropy, *R-measured*. This analysis allows us to examine whether the absorption anisotropy calculated from the isotropic dielectric function is adequate for describing the experimentally measured ensemble anisotropy. The absorption anisotropy is determined by calculating the absorption intensities parallel and perpendicular to the main axis of the nanorod (eqs 4 and 5) using *r* = (μ_||_ – μ_⊥_)/(μ_||_ + 2 μ_⊥_). Because we are considering the isotropic dielectric function for both μ_||_ and μ_⊥_, differences in the absorption intensities along the two axes are solely caused by the attenuation of the electric field due to the anisotropic local field factors, i.e., classical dielectric effects. Emission anisotropy (*q*), on the other hand, is typically determined by measuring the polarized emission intensities along the long and short axes of nanorods through single-particle fluorescence polarization measurements.^47,48,62^ We attempted to determine *q* of single CsPbBr_3_ nanorods by drop-casting a diluted solution of nanorods on a glass slide and measuring the polarized emission intensities (more information can be found in the Supporting Information). However, we note that our sample was unstable under ambient conditions as the PL emission intensity decreased over time (Figure S13), complicating interpretations of emission anisotropy. Nevertheless, as shown by Diroll et al. and others,^47,49,54^*q* can be calculated using eq 2 from the absorption anisotropy and measured ensemble anisotropy at energies far above the band edge. For energies well above the band gap, the contribution of polarized electronic transitions to the absorption anisotropy decreases due to the overlapping of closely spaced electronic states with different transition symmetries. As a result, at high excitation energies, the absorption anisotropy is primarily attributed to dielectric effects. For CsPbBr_3_ nanorods, it can be shown that, at wavelengths below 335 nm, quantum confinement is weak, as the features in the absorption coefficient spectra of nanorods are closely identical with those of bulk CsPbBr_3,_ as mentioned above (Figure 4A). Therefore, we can assume that at 335 nm, the measured anisotropy is mainly governed by dielectric effects, as has been previously demonstrated for cadmium chalcogenide nanorods.^47,49,58^ Under this reasonable assumption, we calculated an emission anisotropy value of *q* = 0.53. We emphasize that this is the characteristic anisotropy for the band edge emission regardless of the excitation energy.

With this fixed emission anisotropy and, separately, the absorption anisotropy calculated using the MG effective medium theory, we calculated *R*-expected according to eq 2. Figure 4C compares the experimentally measured anisotropy with the calculated anisotropy, *R*-expected, determined by ε_isotropic_. We observe at energies well above the band edge that *R*-expected values slightly deviate from the measured anisotropy. However, for energies near the band edge, *R*-expected values significantly deviate from the measured anisotropy. Specifically, *R*-expected calculated from the isotropic dielectric function underestimates the anisotropy at the band edge.

The deviation of *R*-expected from the measured anisotropy is indicative of the distinct electronic contributions to the dielectric function for directions parallel and perpendicular to the nanorod main axis. This dependence is more pronounced at energies near the band edge, where quantum confinement effects strongly affect the electronic structure. In order to determine the anisotropic dielectric function using the IMI method, the absorption coefficient parallel (μ_||_) and perpendicular (μ_⊥_) to the nanorod axis must be known. We developed a procedure to determine the absorption coefficient spectra for directions parallel and perpendicular to the nanorod main axis from the average absorption coefficient spectrum. Our approach involves using the measured anisotropy and previously determined *q* to obtain the experimental absorption anisotropy of nanorod using eq 2. Moreover, since the absorption anisotropy is related to the parallel and perpendicular absorption coefficients through *r* = (μ_||_ – μ_⊥_)/(μ_||_ + 2 μ_⊥_), the experimental absorption anisotropy can be used in conjunction with eq 3 to determine both μ_||_ and μ_⊥_ (further information regarding the procedure and derivation can be found in the Supporting Information).

**Figure 5:**
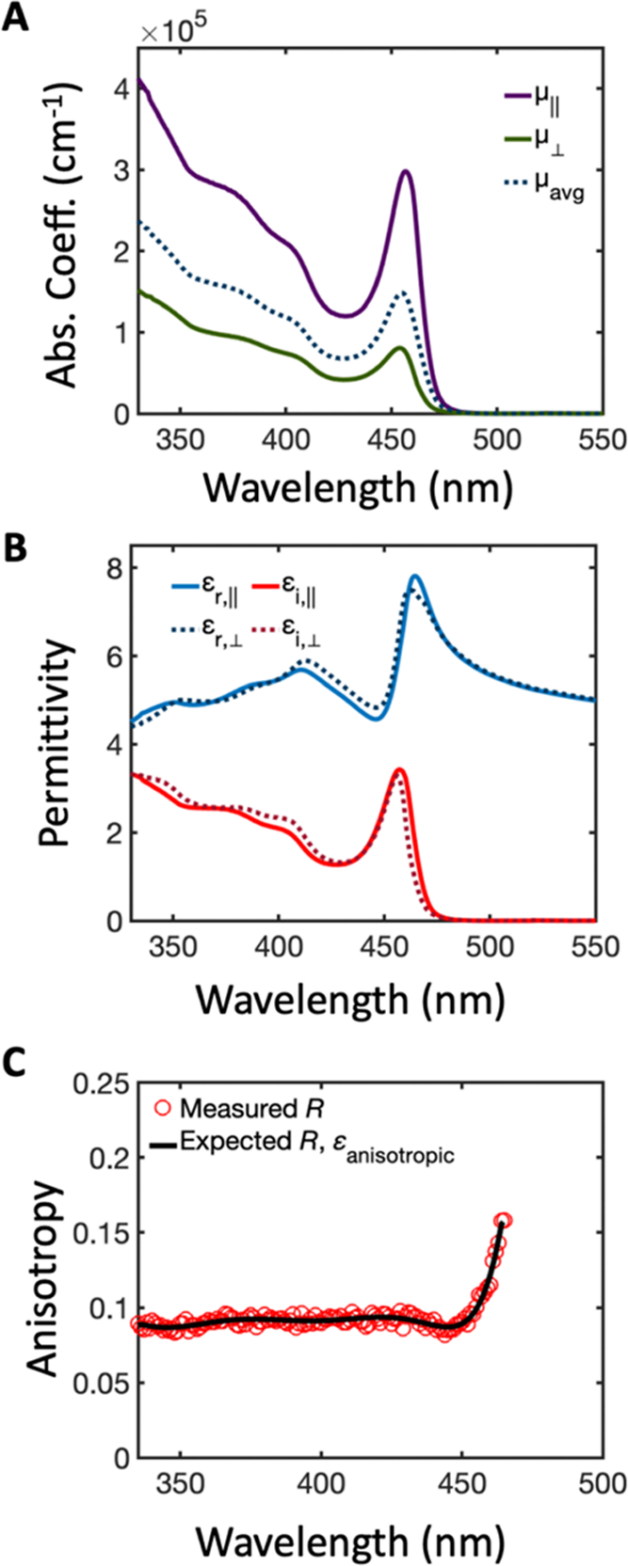
(A) Absorption coefficient spectra for directions parallel (purple) and perpendicular (green) to the nanorod long axis, as determined from the experimental absorption anisotropy and MG effective medium theory. The average absorption coefficient spectrum is shown for reference (dark blue dashed curve). (B) Real and imaginary dielectric portions of the CsPbBr_3_ nanorod for directions parallel (solid lines) and perpendicular (dotted lines) to the nanorod long axis, respectively, found after performing the IMI method. (C) Expected ensemble anisotropy (*R*-expected) calculated using the anisotropic dielectric function.

Figure 5A shows μ_||_ and μ_⊥_ (along with μ_ave_ as determined from ε_isotropic_). We observe an enhancement for μ_||_ relative to μ_ave._, whereas the opposite trend is observed for μ_⊥_. This is expected, in part, due to the different local field factors along each axis. As mentioned above, electric fields parallel to the short axis are screened significantly more compared with electric fields parallel to the long axis, resulting in reduced absorption. Moreover, we found that the relative order and observed relative ratio between all absorption coefficient spectra, particularly at energies far above the band gap, are comparable with previous studies of the absorption coefficient of CdSe nanorods with similar absorption anisotropy.^58^ This observation further indicates that our methodology can appropriately deconvolute the average absorption coefficient into parallel and perpendicular components from the experimental absorption anisotropy.

Having determined the absorption coefficient spectra, we then separately applied the IMI procedure to calculate the complex dielectric functions for directions parallel and perpendicular to the long axis. For this version of the IMI calculation, the theoretical absorption coefficient spectrum of each axis is calculated using eqs 4 and 5. All other parameters in the calculation process are the same as previously described, except for the coefficients in the matrix inversion step, which now depend on the axis under consideration (more information can be found in the Supporting Information). Figure 5B shows the resulting real and imaginary parts of the anisotropic dielectric function (tabulated data are provided in the Supporting Information). In general, similar to the isotropic dielectric function, we observe a blue shift for the first excitonic transition in both ε_I_ spectra that is also accompanied by an increase in the oscillator strength compared to bulk spectra (see Figure S18 for a comparison with experimental values obtained for bulk single-crystal CsPbBr_3_). Moreover, we observe pronounced antiresonances around optical transitions, particularly for the first transition, demonstrating a strong modulation of ε_R_ due to the sharp absorption peaks. Comparing ε_I_ along the two axes, we observe slight changes in both the magnitude and position of spectral features, indicative of the distinct density of states and energies of electronic states along the different axes. Specifically, we observe a slight red shift in the peak position of the first excitonic transition for directions parallel to the nanorod compared to the perpendicular direction. This observation can be understood to arise from the distinct energy levels and selection rules of excitonic transitions within the exciton fine structure of the lowest-energy excitonic state. For instance, a recent study by Folie et al. found that shape anisotropy along with long-range electron–hole exchange interaction effects act to split the exciton band-edge states in quantum-confined 1D CsPbBr_3_ nanowires, resulting in a lowest-energy sublevel polarized along the long axis (*c-*axis) and a manifold of higher-energy states polarized along the short axis.^55^

To further confirm the distinct split and peak shift observed for the lowest-energy transition peak of the complex dielectric function in both parallel and perpendicular directions to the nanorod’s long axis, we have conducted analysis on two additional CsPbBr_3_ nanorod samples with different size dimensions in the quantum confinement regime (see Figure S18–S21 for TEM images, UV–vis diagraming, and size distributions analysis). Consistent with the spectral features depicted in Figure 5B, we observed a comparable splitting and red shift of the peak position of the first excitonic transition for the direction parallel to the nanorod compared to the perpendicular direction (see Figures S22 and S23). These findings further emphasize the significant influence of quantum confinement on the electronic structure and the one-dimensional shape anisotropy on the spectral characteristics of the complex dielectric function in the CsPbBr_3_ nanorods.

To further validate the internal consistency of the resulting anisotropic dielectric function shown in Figure 5B, we calculated the absorption anisotropy using the anisotropic dielectric functions and subsequently calculated *R*-expected using eq 2. We then compared this new *R*-expected value with the measured anisotropy (Figure 5C). As is observed, *R*-expected values closely match the measured anisotropy, providing evidence that the iterative matrix inversion (IMI) calculation is internally consistent with the experimental data. Taken together, these findings collectively demonstrate that the anisotropic dielectric function of CsPbBr_3_ can be determined through a modified IMI procedure by analyzing the absorption anisotropy in conjunction with the ensemble solution-state absorbance spectra. We anticipate that by establishing a comprehensive relationship among absorption spectra, fluorescence anisotropy spectra, and the dimensional characteristics of nanorods possessing different aspect ratios, it will be feasible to utilize particle dimension information obtained from TEM analysis to determine the corresponding dielectric function for new samples.

## Conclusions

We determined the anisotropic complex dielectric function of CsPbBr_3_ nanorods. Our approach uses the ensemble fluorescence anisotropy spectrum in conjunction with the ensemble absorption spectrum to distinguish the absorption of light along the long and short axis of the nanorod. Together, these data enable a modified IMI method for determining the real and imaginary components of the anisotropic dielectric function. Ensemble fluorescence anisotropy measurements show spectrally dependent anisotropy characterized by a peak in anisotropy for band-edge excitation, indicative of the impact of quantum confinement on the electronic structure. Further, the anisotropic complex dielectric function reveals the role of 1D shape anisotropy on features in both the real and imaginary dielectric functions compared with bulk CsPbBr_3_. The findings obtained in this report provide new insights into the optical properties of CsPbBr_3_ nanorods, which will be useful for designing and predicting the efficiency of emerging optoelectronic devices based on this material. Furthermore, the methodology described here can be extended to determine the anisotropic dielectric functions of 1D nanorods with different sizes and compositions.

## Supporting Information Available

Additional TEM images; powder XRD patterns; size distribution histograms; photoluminescence lifetime decay curve; theory of fluorescence anisotropy of nanorods; derivation of equations for the determination of the complex dielectric function; single-particle emission measurements; photoluminescence excitation spectra; tabulated dielectric function values; and fitting procedure of photoluminescence emission spectra (PDF)
