# Supplementary material for: The Anisotropic Complex Dielectric Function of CsPbBr3 Perovskite Nanorods Obtained via an Iterative Matrix Inversion Method
Source: J Phys Chem C Nanomater Interfaces. 2023 Jul 21;127(30):14812–21. doi: 10.1021/acs.jpcc.3c03423 (PMC10863055; doi:10.1021/acs.jpcc.3c03423)
Supplement: Supplementary file 1 — jp3c03423_si_001.pdf [file jp3c03423_si_001.pdf]

Supporting information

The Anisotropic Complex Dielectric Function of $CsPbBr_3$ Perovskite Nanorods Obtained *via* an Iterative Matrix Inversion Method

Freddy A. Rodríguez Ortiz, [a] *Boqin Zhao,* [a] *Je-Ruei Wen,* [a] *Ju Eun Yim,* [a] *Giselle Bauer,* [a] *Anna Champ,* [a] *and Matthew T. Sheldon* [*a,b]

[a] Department of Chemistry, Texas A&M University, College Station, TX 77843, USA

[b] Department of Materials Science and Engineering, Texas A&M University, College Station, TX 77843, USA

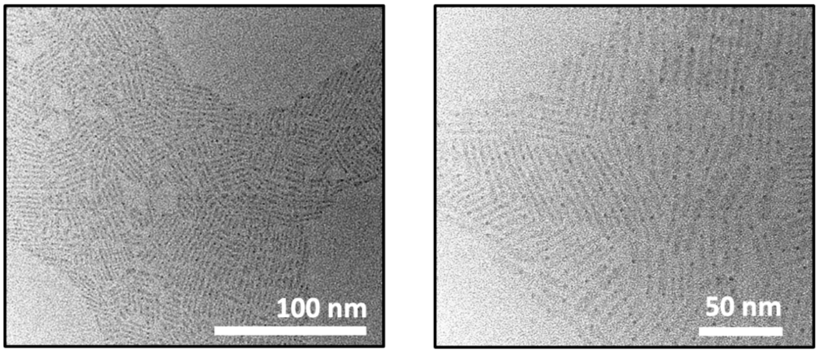

Figure S1. Transmission electron micrographs of $CsPbBr_3$ nanorods.

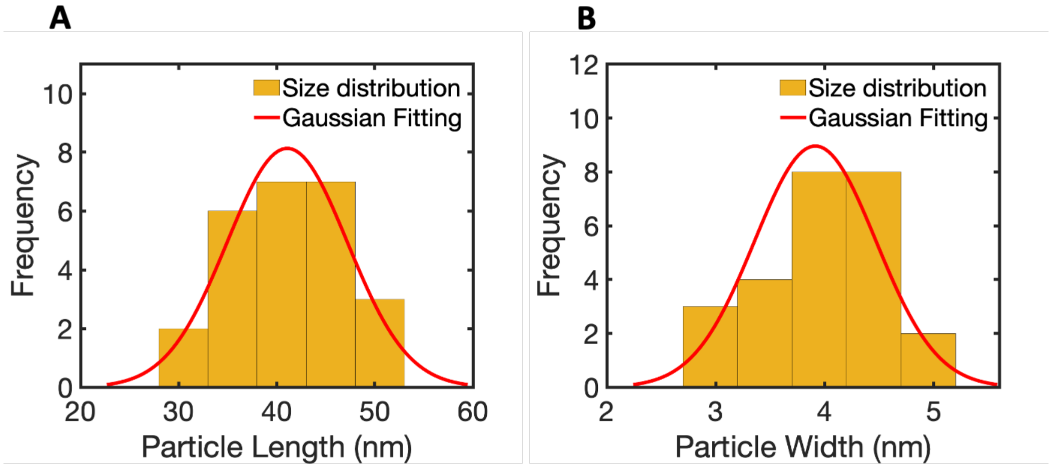

Figure S2. Distribution histograms for $CsPbBr_3$ nanorods (a) length and (b) width fitted with Gaussian functions (sample size = 25 particles). The average length and width were calculated to be (40.2 ± 5.1) nm and (3.91 ± 0.56) nm, respectively.

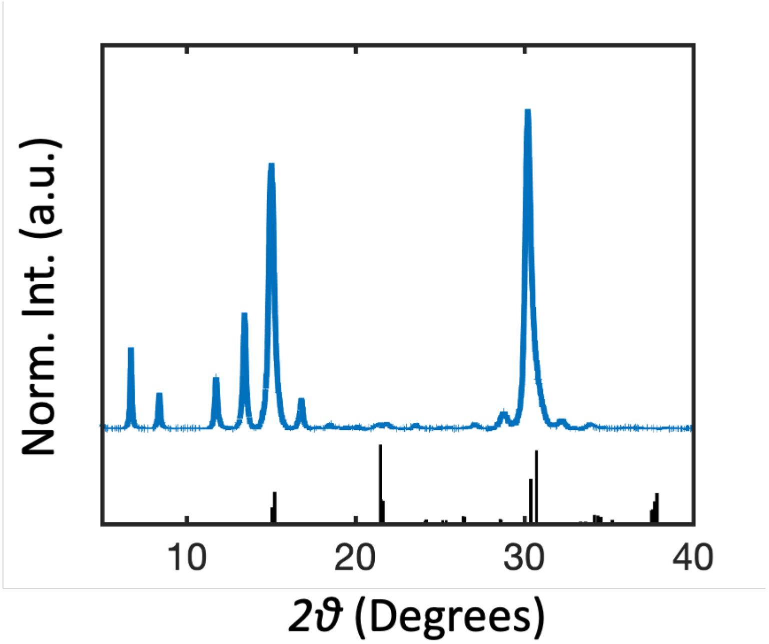

Figure S3. Powder XRD pattern acquired for $CsPbBr_3$ nanorods (blue trace). The diffractogram closely matches the orthorhombic phase (*Pbnm*) of $CsPbBr_3$ (black bars). The reflections in the low angle region ($2\theta < 15º$) correspond to the interparticle distance between nanorods with spacing of ca. 9.2 nm.

Low-energy Localized Emission of the PL Emission Spectra:

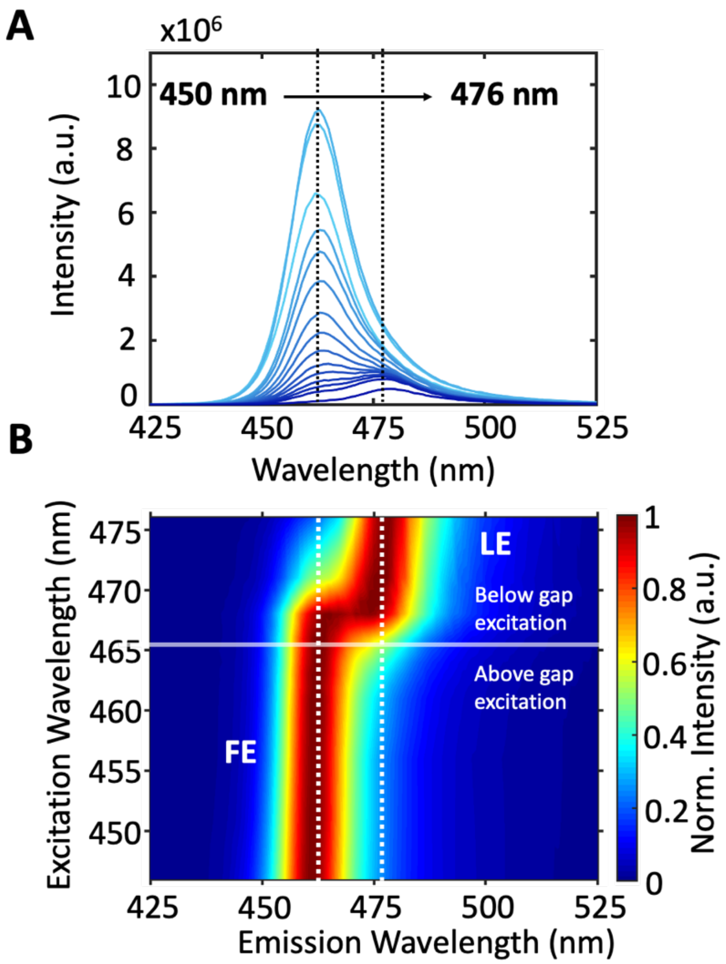

Figure S4. (a) Unnormalized PLE spectra of a solution of CsPbBr3 nanorods in the excitation range of 450 nm to 476 nm. (b) Normalized 2D PLE map of spectra in (a). The left and right vertical dotted lines in (a-b) denote the emission peaks corresponding to the free exciton emission and localized exciton emission, respectively.

To investigate the low-energy tail of the PL emission spectra, we performed photoluminescence excitation spectroscopy (PLE) measurements on an ensemble solution of $CsPbBr_3$ nanorods. For this, we monitored the PL emission as a function of excitation

wavelength for both above and below the nanorod bandgap. Figure S4a-b display the unnormalized PLE spectra and normalized 2D PLE map acquired for a solution of $CsPbBr_3$ nanorods at room temperature. For above bandgap excitations, a feature centered at 463 nm is apparent, which can be attributed to near band-edge radiative recombination from free excitons. Interestingly, as the excitation wavelengths move towards lower energies below the absorption edge, an additional feature centered at 476 nm increases in intensity until eventually becoming the dominant feature. We denoted this feature as localized exciton (LE) emission as the spectral position of the emission peak remains constant regardless of the excitation wavelength.

To gain further understanding of the origin of LE emission, we conducted time-resolved photoluminescence decay measurements by exciting an ensemble solution of nanorods at 435 nm and fixing the detection wavelength at the center of each peak. As shown in Figure S5, the PL decay curves exhibit distinct decay rates. To determine the lifetime values, we fitted each PL decay curves to a biexponential decay function: $I = I_0 + \alpha_1 e^{-t/\tau_1} + \alpha_2 e^{-t/\tau_2}$ where, *I* is the emission intensity, τ_1, τ_2 are the characteristic lifetime values, and α_1, α_2 represent the relative contribution of each one of the components to the total emission. The average decay lifetime of FE emission (18 ns) is significantly faster than LE emission (120 ns). It is interesting to note that both FE and LE emission possess a similar fast decay component, approximately 2.4 ns, however, they differ in their long decay component values. The τ_2 value for FE emission is 42 ns, whereas for LE emission it is 136 ns. The energy splitting between the FE and LE peak was calculated to be 82 meV.

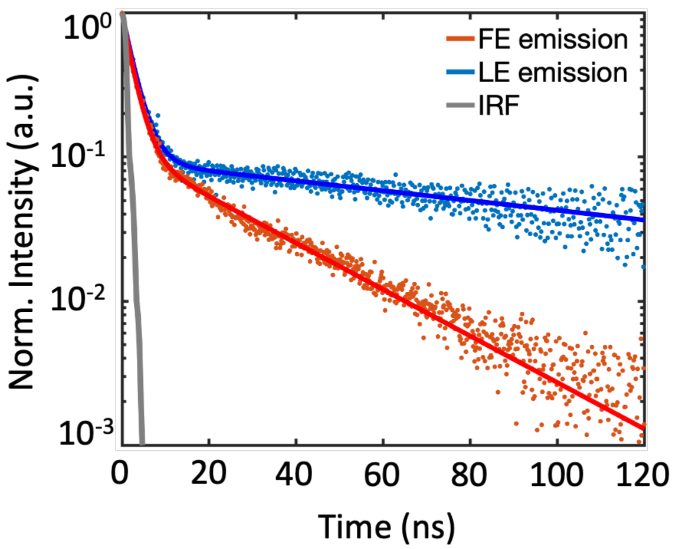

Figure S5. Time resolved photoluminescence decay curves at room temperature monitored at 463 nm (red dots) and 476 nm (blue dots). The lifetime values were extracted by fitting the PL decay curves with biexponential functions (solid traces).

We have found several proposed mechanisms for the low-energy tail in the PL spectra of $CsPbBr_3$ nanocrystals, including size polydispersity and emission from extrinsic defect states, dark exciton, and self-trapped exciton (STE). Here, low-energy emission from size polydispersity, below-gap trap defects or dark exciton were ruled out by examining the spectral characteristics of the absorption and PL emission spectra, such as lifetime and energy splitting of the LE peak relative to the FE peak. Size polydispersity and presence of below-gap in nanocrystals typically manifest as a long red tail in the absorption spectra. However, we observed a well-defined and sharp absorption edge for our samples, suggesting that these factors are unlikely to be the origin of the LE emission. This is further supported by electron microscopy observations (Figure 1a-b) which indicate a high degree of nanorod size uniformity. Moreover, lower energy emission from dark excitons typically exhibits longer lifetimes (1.2 μs) and smaller energy splitting (20 meV) than what is observed in this study. Rather, our results

align more closely with previous studies on self-trapped exciton (STE) emission, which occurs when free excitons are trapped by local deformation of the crystal lattice due to strong electron-phonon coupling. Previous studies have reported STE lifetimes and energy splitting similar to what is observed in this study. For instance, a study by Ma et al. on $CsPbBr_3$ nanocrystals demonstrated similar PL decay dynamics for both FEs and STEs. It was observed that the fast component of the STE decay was similar to that of the FE decay, while the slow components were different, consistent with the observations in this study. They attributed the fast component of the STE decay to the thermal-induced transformation of STEs into FEs, and the slow component of the FE peak decay to FEs arising from STEs. In light of these findings, we hypothesis that the low-energy emission in our sample is likely arising from STEs, although further experiments are required to confirm this.

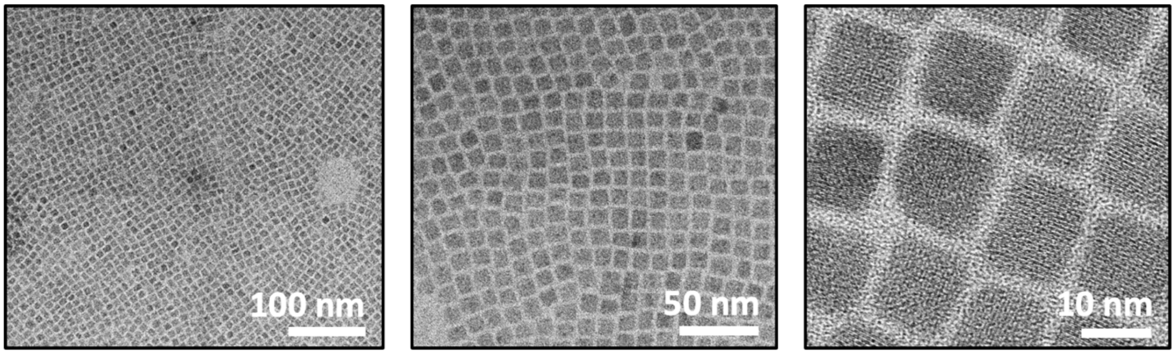

Figure S6. Transmission electron micrographs of $CsPbBr_3$ nanocubes.

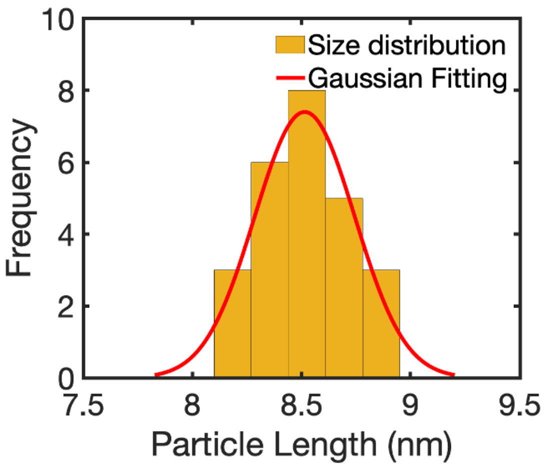

Figure S7. Size distribution histogram for cuboid shaped $CsPbBr_3$ nanocrystals fitted with a Gaussian function (sample size = 25 particles). The average length was calculated to be 8.51 ± 0.23 nm.

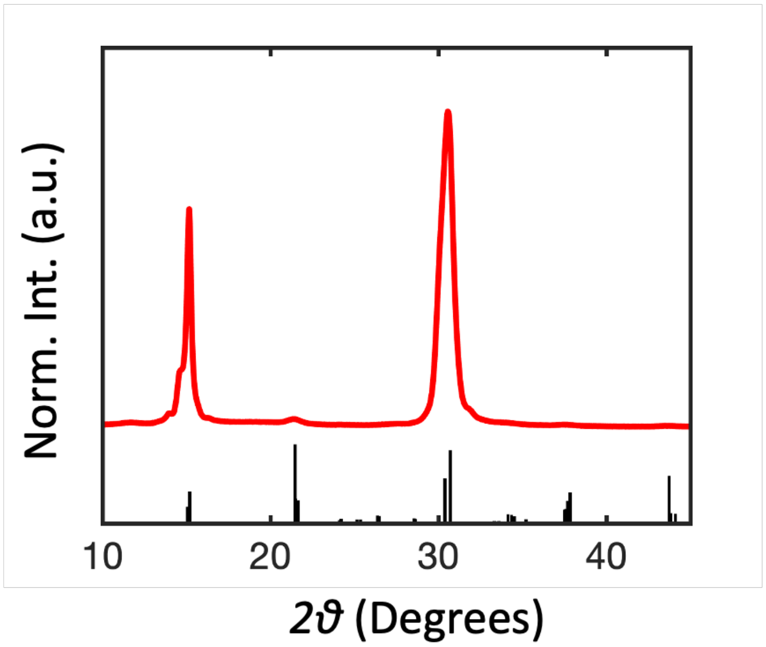

Figure S8. Powder XRD pattern acquired for $CsPbBr_3$ nanocubes (red trace). The diffractogram closely matches the orthorhombic phase (*Pbnm*) of $CsPbBr_3$ (black bars).

Accuracy of Optical Anisotropy Measurements

For small fluorophore in low viscosity solvents, the rate of rotational diffusion (i.e., Brownian rotation) is typically faster than the rate of fluorescence emission, leading to depolarization of fluorescence. Although prominent for dichroic dyes, the effect of rotational diffusion on the optical anisotropy of nanorods is less significant. Characteristic rotational times of nanorods vary by size and viscosity of the solvent, but these values are greater than 1 μs, even in low-viscosity solvents at room temperature.[1, 2] Fluorescence from $CsPbBr_3$ nanorods, however, is significantly faster than the rotational times of nanorods with a measured average fluorescence lifetime of 18 ns (Figure S9). Furthermore, several previous reports on optical anisotropy of nanorods show that dispersing solvent minimally changes the magnitude of optical anisotropy, if at all.

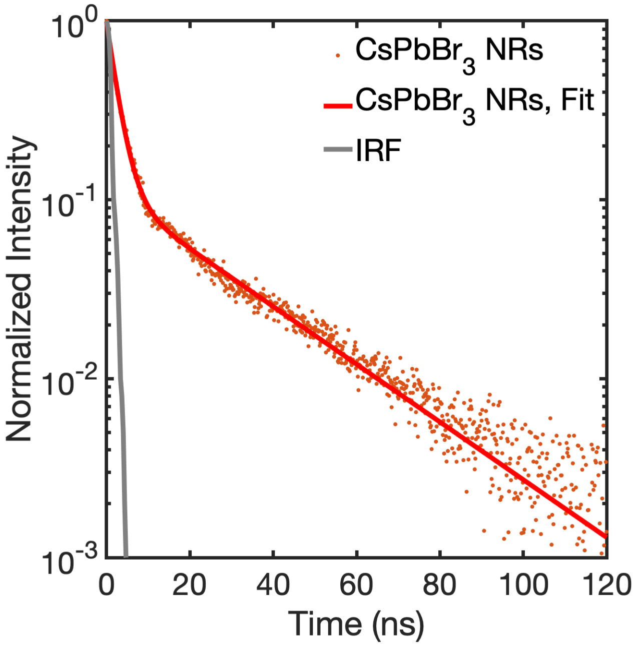

Figure S9. Time resolved photoluminescence decay curve at room temperature for $CsPbBr_3$ nanorods (red dots). The instrument response factor (IRF) is shown in gray. The average PL lifetime value after fitting the PL decay with a biexponential function was calculated to be 18 ns.

Theory of Fluorescence Anisotropy of Nanorods

A complete description regarding the theory of fluorescence anisotropy can be found in refs.[3] Traditionally, the analytical expression of anisotropy is derived from the angular displacement of the absorption and emission transition dipoles, according to:

$$R = \frac{2}{5}\left(\frac{3\cos^2\beta - 1}{2}\right) \qquad \text{(S1)}$$

where the 2/5 prefactor describes the loss of anisotropy due to photoselection, and β represents the angle between the absorption and emission transition dipoles.[3] This approach, which is common for molecular fluorophores, presupposes that the absorption and emission transition

dipoles are simple linear dipole oscillators. However, this is not the case for optical transitions in nanorods. The polarization of both absorption and emission in nanorods are dictated by the cylindrical/ellipsoidal symmetry, resulting in a z-component along the nanorod with photons linearly polarized, and equal x and y components perpendicular to the nanorod with photons planar polarized in the ab plane.[4] Because of the spectroscopic properties of nanorods, the relative distribution of absorption and emission intensities along these axes determines the fluorescence anisotropy. Determination of fluorescence anisotropy from the absorption and emission intensities can be found in the existing literature with a thorough derivation and application to nanorods performed by Sitt *et al*.[5] For convenience, the work of Sitt *et al* is briefly

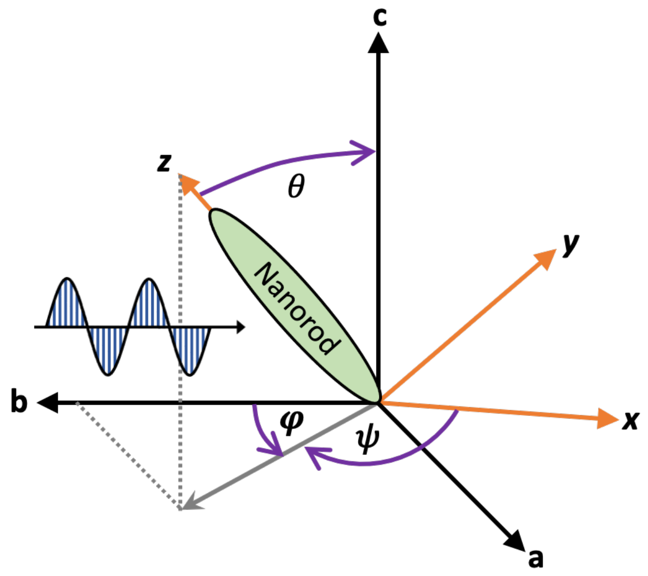

Figure S10. Diagram indicating the laboratory and rod coordinates system. The laboratory coordinates (in black): a, the emission collection path; b, the excitation light path; and c, the polarization direction. The rod coordinates system (in orange): z, the rod main axis and xy, the plane perpendicular to the main axis. $\boldsymbol{\theta}$, $\boldsymbol{\varphi}$, and $\boldsymbol{\psi}$ depict the Eulerian angles.

discussed here:

In the photoselection experiment of nanorods, shown in Figure S10, the sample is illuminated with vertical polarized light along the laboratory fixed b axis. The nanorods are assumed to be randomly distributed in the sample such that the nanorod fixed axes x, y, and z (shown in orange) are randomly oriented relative to the laboratory axes (shown in black). Upon illumination, those nanorods with absorption transition dipoles aligned parallel to the electric vector of the polarized light will have the highest probability of excitation due to photoselection, resulting in a population of nanorods that are partially oriented along the laboratory c axis. The probability of absorption is proportional to the square of the projection of the absorbing transition dipole moment onto the polarization axis of the excitation light (c-axis) and is described by $r_x = r_y < r_z$ along the x, y, or z axis of the nanorod, respectively. Similarly, the probability that the emission transition occur along a specific orientation is proportional to the square of the projection of the emission transition dipole moment on the orientation axis and is describe by $q_x = q_y < q_z$ along the x, y, or z axis of the nanorod, respectively. These probabilities are normalized such that $r_x + r_y + r_z = 1$ and $q_x + q_y + q_z = 1$. Because of the symmetry of the nanorod geometry, the components of the intensity along the minor axes ($r_x = r_y$ and $q_x = q_y$) are assumed to be equivalent. The probability of absorption and emission events occurring can be determined by the product of the individual probabilities of each event as described below.

The orientation of the nanorod with respect to the laboratory axes can be specified using the Eulerian angles (Figure S10). The transformation matrix from the nanorod coordinates to the laboratory coordinates, A, is given by:

$$A = \begin{bmatrix} A_{ax} & A_{ay} & A_{az} \\ A_{bx} & A_{by} & A_{bz} \\ A_{cx} & A_{cy} & A_{cz} \end{bmatrix} \tag{S2}$$

where

$A_{ax} = \cos\theta$,

$A_{ay} = \sin\theta \sin\psi$,

$A_{az} = \sin\theta \cos\psi$,

$A_{bx} = \sin\theta \sin\varphi$,

$A_{by} = \cos\psi \cos\varphi - \cos\theta \sin\psi \ \sin\varphi$,

$A_{bz} = - \ \sin\psi \cos\varphi - \ \cos\theta \cos\psi \sin\varphi$,

$A_{cx} = - \sin\theta \cos\varphi$,

$A_{cy} = \cos\psi \sin\varphi + \cos\theta \sin\psi \ \cos\varphi$,

$A_{cz} = - \ \sin\psi \sin\varphi + \ \cos\theta \cos\psi \cos\varphi$

The absorption probability for vertically polarized light (traveling along the laboratory b axis and polarized along the laboratory c axis) is given by:

$$P_{Abs} = \ r_x A_{cx}^2 + r_y A_{cy}^2 + r_z A_{cz}^2 = \frac{1}{2} \ (1 - r_z)\left(A_{cx}^2 + A_{cy}^2\right) + r_z A_{cz}^2 \ , \qquad \text{(S3)}$$

Similarly, the emission probability along the *l* axis (*l* = *b, c*) is:

$$P_{Em}^l = \ q_x A_{lx}^2 + q_y A_{ly}^2 + q_z A_{lz}^2 = \frac{1}{2} \ (1 - q_z)\left(A_{lx}^2 + A_{ly}^2\right) + q_z A_{lz}^2 \ , \qquad \text{(S4)}$$

The averaged emission intensity of randomly oriented nanorods along the polarization l axis can be obtained by integrating over all Eulerian angles, according to:

$$I_l \propto \int_0^{2\pi} d\psi \int_0^{2\pi} d\varphi \int_0^{\pi} P_{Abs} \, P_{Em}^l \sin\theta \, d\,\theta \qquad \text{(S5)}$$

With θ, φ, and ψ being the Eulerian angles. The optical anisotropy of the sample can be calculated according to:

$$R = \frac{I_c - I_b}{I_c + 2I_b} \qquad \text{(S6)}$$

As it is further discussed in the report of Sitt *et al*,[5] by using this procedure, it is possible to map the degree of polarization of single nanorod to the ensemble anisotropy. A similar procedure was also reported by Diroll *et al.*,[6] which allows the determination of the ensemble anisotropy from (1) known projection of absorption and emission intensity onto the nanorod unique axis or (2) known absorption and emission anisotropy of nanorod, according to:

$$R = \frac{2}{5}\left(\frac{3r_z - 1}{2}\right)\left(\frac{3q_z - 1}{2}\right) = \frac{2}{5}\left(\frac{r_{||} - r_{\perp}}{r_{||} + 2r_{\perp}}\right)\left(\frac{q_{||} - q_{\perp}}{q_{||} + 2q_{\perp}}\right) = \frac{2}{5} * r * q \tag{S7}$$

Here, the 2/5 prefactor arises from photoselection, the $(3r_z - 1)/2$ and $(3q_z - 1)/2$ terms represent the average projection of the absorption and emission intensities onto the z axis of the nanorod, and $(r_{||} - r_{\perp})/(r_{||} + 2r_{\perp})$ and $(q_{||} - q_{\perp})/(q_{||} + 2q_{\perp})$ represent the absorption anisotropy and emission anisotropy of nanorod, respectively. Figure S11 displays the expected anisotropy using photoselection for given absorption and emission anisotropy values, reproduced from Diroll *et al.* [6]

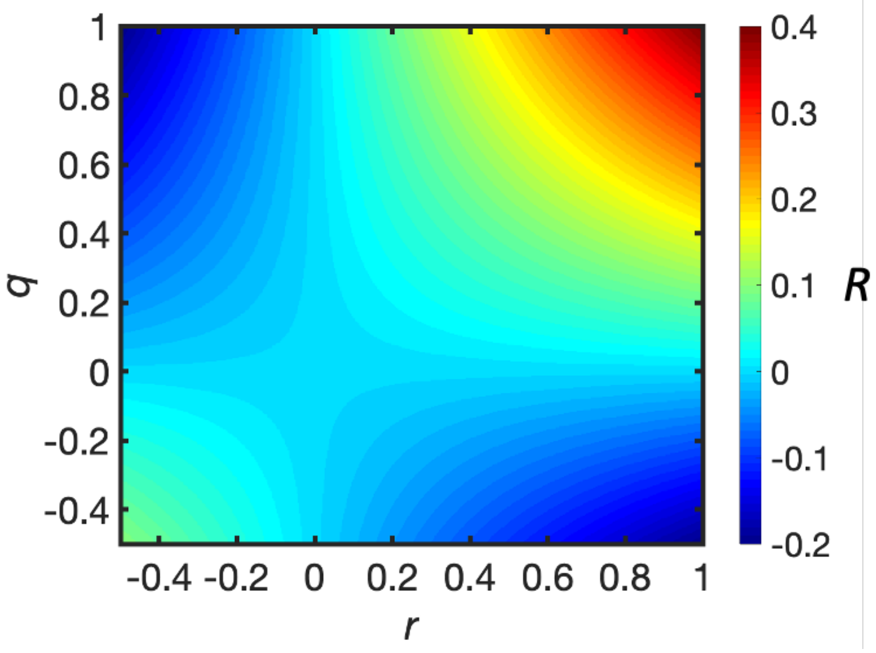

Figure S11. Contour plot of the expected value of anisotropy (R) as a function of the single particle absorption (r) and emission (q) anisotropy.

Calculation of the Complex Dielectric Function of $CsPbBr_3$ Nanorod *via* Iterative Method

To calculate the complex dielectric functions of $CsPbBr_3$ nanorods, we modified an iterative matrix inversion (IMI) method by deriving expressions to account for the nanorod morphology. Using this modified method, we were able to 1) calculate the average complex dielectric function for the nanorod from the average absorption coefficient spectrum and 2) determine the complex dielectric functions for directions parallel and perpendicular to the nanorod main axis from their respective absorption coefficient spectra. In this section, we show the derivation of expressions for the calculation of both the average and anisotropic dielectric functions.

1. **Theoretical Expressions and Discussion for the Calculation of the Average Dielectric Function of Nanorod**

A. **Maxwell-Garnett Effective Medium Theory for Colloidal Nanorods**

As discussed in the main text, when randomly dispersed colloidal nanorods are modeled as prolate ellipsoids of rotation, their absorption coefficient ($\mu_{i,NR}$) becomes a weighted average over all the rod orientations.[7] Since an ellipsoid has only one parallel but two perpendicular directions, this yields:

$$\mu_{i,NR(ave.)} = \frac{1}{3}\,\mu_{i,||} + \frac{2}{3}\,\mu_{i,\perp} \tag{S8}$$

Where,

$$\mu_{i,||} = \frac{2\pi}{\lambda n_s}\left|f_{LF,||}\right|^2 \varepsilon_{I,||} = \frac{2\pi}{\lambda n_s}\frac{{n_s}^4}{\left(\alpha_{||}\,\varepsilon_{R,||}+(1-\alpha_{||})n_s{}^2\right)^2+\left(\alpha_{||}\,\varepsilon_{I,||}\right)^2}\;\varepsilon_{I,||} \tag{S9}$$

$$\mu_{i,\perp} = \frac{2\pi}{\lambda n_s}\left|f_{LF,\perp}\right|^2 \varepsilon_{I,\perp} = \frac{2\pi}{\lambda n_s}\frac{{n_s}^4}{\left(\alpha_{\perp}\,\varepsilon_{R,\perp}+(1-\alpha_{\perp})n_s{}^2\right)^2+\left(\alpha_{\perp}\,\varepsilon_{I,\perp}\right)^2}\;\varepsilon_{I,\perp} \tag{S10}$$

Here, $f_{LF,||}$ and $f_{LF,\perp}$ denote the parallel and perpendicular local field factors, n_s is the refractive index of the surroundings, $\alpha_{||}$ and $\alpha_{\perp}$ are the parallel and perpendicular depolarization factors, and $\varepsilon_{R,||}$, $\varepsilon_{R,\perp}$ and $\varepsilon_{I,||}$, $\varepsilon_{I,\perp}$ are the real and imaginary parts of the dielectric function for directions parallel and perpendicular to the long axis of the nanorod, respectively.

The depolarization factors for a prolate ellipsoid[8, 9] with a major semi-axis x and minor semi-axes y and z ($a_x > a_y = a_z$) can be calculated according to:

$$\alpha_{||} = \frac{1-e^2}{2e^3}\left(ln\frac{1+e}{1-e} - 2e\right) \quad \text{(S11)}$$

$$\alpha_{\perp} = \frac{1}{2}\left(1 - \alpha_{||}\right) \quad \text{(S12)}$$

where the eccentricity (e) is:

$$e = \sqrt{1 - \frac{a_y^2}{a_x^2}} \quad \text{(S13)}$$

The three depolarization factors for any ellipsoid satisfy: $\alpha_x + \alpha_y + \alpha_z = 1$. Modelling $CsPbBr_3$ nanorods as prolate ellipsoid with a major axis of 40 nm and a minor axis of 4 nm, we obtain $\alpha_{||}$ = 0.0202 and $\alpha_{\perp}$=0.4898.

B. Discrete Kramers-Krönig Relations

A detailed explanation can be found in the work of Moreels *et al.*[10] For convenience, we have reproduced some expression and discussion from their work. The real and imaginary parts of the dielectric function are related through Kramers-Krönig (KK) relations, according to (P = Cauchy principal value):

$$\varepsilon_R(\omega) = 1 + \frac{2}{\pi} P\int_0^{\infty} \frac{\omega'\varepsilon_I(\omega')}{\omega'^2 - \omega^2}\, d\omega' \quad \text{(S14)}$$

From this relation, ε_R at a given frequency (ω) can be calculated from an integration of ε_I over the entire frequency domain. However, the absorption spectrum of nanocrystals is typically

measured over a wavelength range, rather than a frequency range, and it is determined at discrete, evenly spaced wavelengths instead of a continuous range. Using $\omega \cdot \lambda = 2\pi \cdot c$ (c = speed of light), we can transform equation S14 into the wavelength domain. Additionally, we can rewrite equation S14 in a discrete from:

$$\varepsilon_R(\lambda_j) = 1 + \frac{2}{\pi}\sum_{k\neq j}\frac{\lambda_j^2 \Delta\lambda}{\lambda_k(\lambda_j^2 - \lambda_k^2)}\varepsilon_I(\lambda_k) \tag{S15}$$

The summation runs from $k = 0$ to ∞, omitting $k = j$, thus avoiding infinite values. This is equivalent to using the Cauchy principal value in the continuous KK relation. Considering that $\lambda_{j(k)} = j(k) \cdot \Delta\lambda$, equation S15 can be simplified to:

$$\varepsilon_{R,j} = 1 + \frac{2}{\pi}\sum_{k\neq j}\frac{j^2}{k(j^2 - k^2)}\varepsilon_{I,k} \tag{S16}$$

Moreover, equation S15 can be written using a matrix formalism by writing ε_R and ε_I as column vectors. In addition, since our experimental bulk permittivity data only extended to 230 nm, we replace 1 by ε_∞, following similar studies.[10, 11] This term approximates the effect of higher lying transitions features not observed in the experimental imaginary part of the dielectric function of bulk $CsPbBr_3$. The value of ε_∞ was determined by taking the difference between KK consistent permittivity value and that of the real permittivity of bulk $CsPbBr_3$ at 335 nm, following the approach of Dement *et al.*[11] The resulting value for ε_∞ was 2.8.

$$\varepsilon_R = \varepsilon_\infty + \frac{2}{\pi} A \cdot \varepsilon_I \tag{S17}$$

with

$$A_{j,k} = \frac{j^2}{\mathrm{k}(j^2 - k^2)}, \qquad A_{j,j} = 0$$

C. Method to Calculate the Average Nanorod Dielectric Function: Iterative Matrix Inversion (IMI)

Returning to the expression in S8-S10, MG theory implies that the absorption coefficient is dependent on both the real and imaginary part of the dielectric function. Therefore, a straightforward calculation of optical constants as for bulk materials is not possible. This issue can be circumvented by applying an iterative procedure to calculate the dielectric function (Figure 3). The iterative procedure involves comparing the experimentally determined intrinsic absorption coefficient spectrum, $\mu_k^{(exp.)}$, to the calculated absorption coefficient spectrum, μ_k, (i.e., μ spectrum determined at each iteration by varying the permittivity values), until μ_k converges to $\mu_k^{(exp.)}$, which gives the real and imaginary permittivity functions. The iterative process is described as follows.

Starting from an initial guess for the imaginary part of the dielectric function ($\varepsilon_{I,0}$), we calculate the corresponding $\varepsilon_{R,0}$ *via* KK relations. We used the discrete form of the KK integral (equation S15) to transform new trial functions of the imaginary permittivity into the corresponding KK real permittivity functions, ensuring that the subsequent trial function remained KK consistent. For our initial guess function, we used the experimental bulk values of $CsPbBr_3$ determined using spectroscopic ellipsometry by Mannino *et al.*[12] In addition, as it is further discussed in the text, we assume bulk values at short wavelengths (below 335 nm). These initial functions then yield an initial theoretical estimate for the absorption coefficient ($\mu_{k,0}$) using equation S8, with $\mu_{k,0} \neq \mu_k^{(exp.)}$. We then obtain a new trial function for ε_I by calculating a first-

order correction of $\varepsilon_{I,0}$ through linearization of the ratio $\mu_{k,0}$ / $\mu_k^{(exp.)}$. For this, we define $\Delta\varepsilon_I$ and $\Delta\varepsilon_R$ as the difference between the initial trial function and the true values ($\varepsilon_I = \varepsilon_{I,0} + \Delta\varepsilon_I$, $\varepsilon_R = \varepsilon_{R,0} + \Delta\varepsilon_R$). Substituting these functions in equation S8-S10, a first-order Taylor series expansion yields (using the matrix notation):

$$\mathbf{M} = \mathbf{C}\Delta\boldsymbol{\varepsilon}_\mathbf{I} + \mathbf{D}\Delta\boldsymbol{\varepsilon}_\mathbf{R} \tag{S18}$$

With

$$\mathrm{M_j} = \frac{\mu}{\mu_0} - 1$$

$$\mathrm{C_{j,j}} = \frac{\frac{1}{\sigma_a} + \frac{2}{3\sigma_b} - \frac{2\alpha_{||}^2\,\varepsilon_I^2}{3\left(\left(\alpha_{||}\,\varepsilon_R - n_s^2(\alpha_{||}-1)\right)^2 + \alpha_{||}^2\,\varepsilon_I^2\right)^2} - \frac{4\alpha_\perp^2\,\varepsilon_I^2}{3\sigma_b^2}}{\frac{\varepsilon_I}{\sigma_a} + \frac{2\varepsilon_I}{3\sigma_b}}$$

Where

$$\sigma_a = 3\left(\left(\alpha_{||}\,\varepsilon_R - n_s^2(\alpha_{||}-1)\right)^2 + \alpha_{||}^2\,\varepsilon_I^2\right)$$

$$\sigma_b = \left(\alpha_\perp\,\varepsilon_R - n_s^2(\alpha_\perp-1)\right)^2 + \alpha_\perp^2\,\varepsilon_I^2$$

And,

$$\mathrm{D_{j,j}} = -\frac{\frac{2\alpha_{||}\,\varepsilon_I\left(\alpha_{||}\varepsilon_R - n_s^2(\alpha_{||}-1)\right)}{3\sigma_d^2} + \frac{4\alpha_\perp\,\varepsilon_I(\alpha_\perp\,\varepsilon_R - n_s^2(\alpha_\perp-1))}{3\sigma_c^2}}{\frac{\varepsilon_I}{3\sigma_d} + \frac{2\varepsilon_I}{3\sigma_c}}$$

Where

$$\sigma_c = (\alpha_{\perp}\varepsilon_R - n_s{}^2(\alpha_{\perp} - 1))^2 + \alpha_{\perp}{}^2\,\varepsilon_I{}^2$$

$$\sigma_d = \left(\alpha_{||}\,\varepsilon_R - n_s{}^2\left(\alpha_{||} - 1\right)\right)^2 + \alpha_{||}{}^2\,\varepsilon_I{}^2$$

$$C_{j,k} = D_{j,k} = 0$$

The difference of the imaginary part of the dielectric function ($\Delta\varepsilon_I$) between the initial trial function and true values can be calculated by matrix inversion:

$$\Delta\boldsymbol{\varepsilon}_\mathbf{I} = [\mathbf{C} + (\mathbf{2}/\boldsymbol{\pi})\mathbf{DA}]^{-\mathbf{1}}\mathbf{M} \tag{S19}$$

where **A** already defined in equation S17. The corrected trial function ($\varepsilon_{I,1} = \varepsilon_{I,0} + \Delta\varepsilon_I$) is then used in the next iterative step. Generally, the dielectric function values obtained after one iteration step do not result in $\mu_k = \mu_k^{(exp.)}$. However, by iterating the procedure described above, the absorption coefficient calculated at each iterative step μ_k will gradually converge to $\mu_k^{(exp.)}$. The iteration process is ceased once the root-mean-square error (RMSE) is reduced to values below 10^{-6}, as described in the text. For $CsPbBr_3$ nanorod, this was achieved in 3 iterative steps (Figure S12).

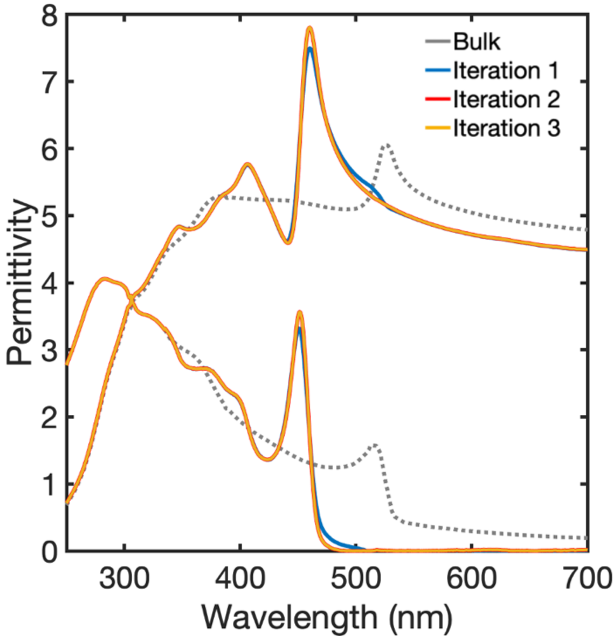

Figure S12. Real and imaginary parts of the permittivity of $CsPbBr_3$ nanorod determined through the iterative procedure after 1, 2, and 3 iteration steps. The bulk permittivity values for $CsPbBr_3$ are shown in gray (dotted line).

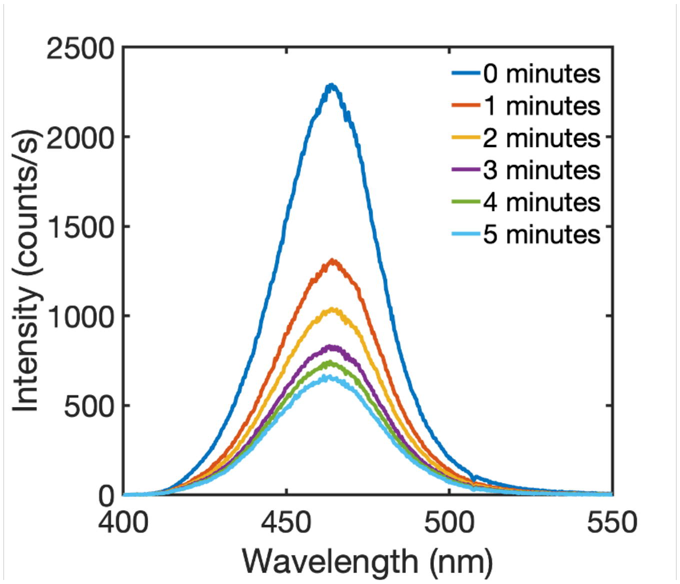

Figure S13. PL emission spectra acquired for $CsPbBr_3$ nanorods deposited on a microscope glass slide at various times.

Single-particle Emission Measurements

PL emission spectra measurements were carried out by dropcasting a dilute solution of nanorods on a microscope slide and using a 405 nm laser (RGBLase FBB-405-200-FS-C-1-0) coupled through free space into a confocal microscope system (Witec RA300). The excitation beam was vertically polarized using a hand-mounted Glan-Taylor polarizer (Thorlabs GT15-A). The emission was collected by a 100x objective (Zeiss EC Epiplan Neofluar, NA = 0.9, WD = 0.31mm), polarized through a linear glass polarizer (Edmund 47-316) inserted in the emission channel, and detected using an ultra-high throughput spectrometer (UHTS300, grating = 300 g/mm). Each emission spectra were integrated for 0.5 second. Data acquisition was performed using commercial software (Witec Control FOUR). Figure S13 shows the PL emission spectra of $CsPbBr_3$ nanorods collected at various times.

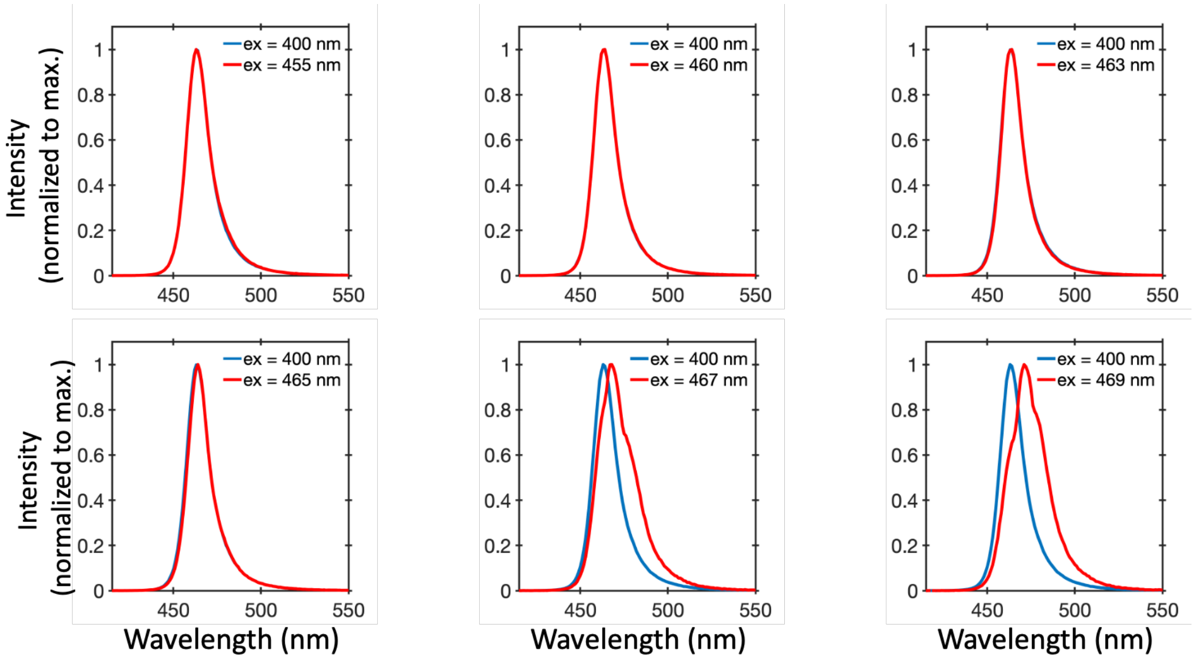

Figure S14. PL emission spectra of $CsPbBr_3$ nanorods acquired at various excitation wavelength for vertical excitation and vertical emission polarization (red traces). The PL emission spectra are compared to the PL emission spectrum obtained at 400 nm (blue traces), where scattering of excitation light does not affect the intensity or spectral shape of the nanorod emission. PL emission spectra of $CsPbBr_3$ NRs acquired at excitation energies above 465 nm show evidence of light scattering from the excitation light (e.g., PL emission spectra acquired at 467 nm and 469 nm excitation).

Calculation of the Anisotropic Dielectric Function of Nanorod

A. Determination of the absorption coefficient parallel and perpendicular to the long axis of the nanorod

We have developed an alternative approach for determining the complex dielectric function along the main and short axes of the nanorod. The method involves using the fluorescence anisotropy of ensembles with the average absorption coefficient spectrum (Figure 4a) to

determine the absorption coefficient spectra along the two axes, which can then be used with the iterative method to calculate the real and imaginary part of the dielectric functions.

First, to use the fluorescence anisotropy to obtain the absorption coefficient spectra along the two axes, it is necessary to have the full anisotropy spectrum. However, fluorescence anisotropy of nanocrystals using photoselection is typically measured for excitation above the bandgap, as the absorption of nanocrystals decreases exponentially for excitation energies below the band gap. In the main text (Figure 2), the fluorescence anisotropy of $CsPbBr_3$ nanorods was measured in the wavelength range of 335 nm to 463 nm. We extended the fluorescence anisotropy spectrum for wavelengths above 463 nm and below 335 nm. For wavelengths above 463 nm, we measured the anisotropy by exciting nanorods at the Urbach tail and recording the PL emission spectra with polarization parallel (I_{VV}) and perpendicular (I_{VH}) to the excitation. It is worth noting that a challenge in these measurements was the scattering of the excitation by the sample for below band-gap excitations, particularly when both the excitation and emission polarizers are coaligned (I_{VV}). This scattering can be observed in the PL emission spectra (I_{VV}) as an additional peak centered at the excitation wavelength (Figure S14)

To overcome this issue, we employed a fitting procedure utilizing Voigt functions to deconvolute the scattering peak from the PL emission spectra, and to extract the peak intensities corresponding to the FE peak. The extracted FE peak intensities were then utilized to calculate the fluorescence anisotropy according to equation 1. Further information on the fitting procedure can be found below, including an example of a fitted PL emission spectrum in Figure S18.

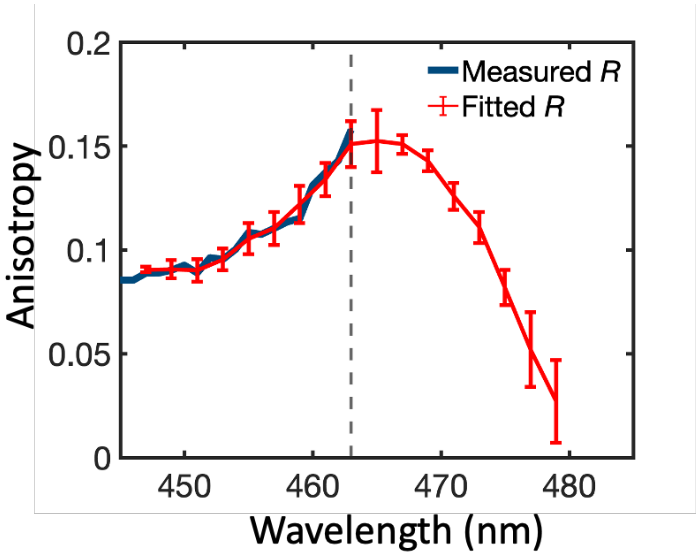

Figure S15. Measured fluorescence anisotropy (blue curve) for excitation wavelengths above the emission peak and fitted fluorescence anisotropy (red curve) for excitation wavelengths above and below the emission peak. The vertical dashed line denotes the emission peak maximum.

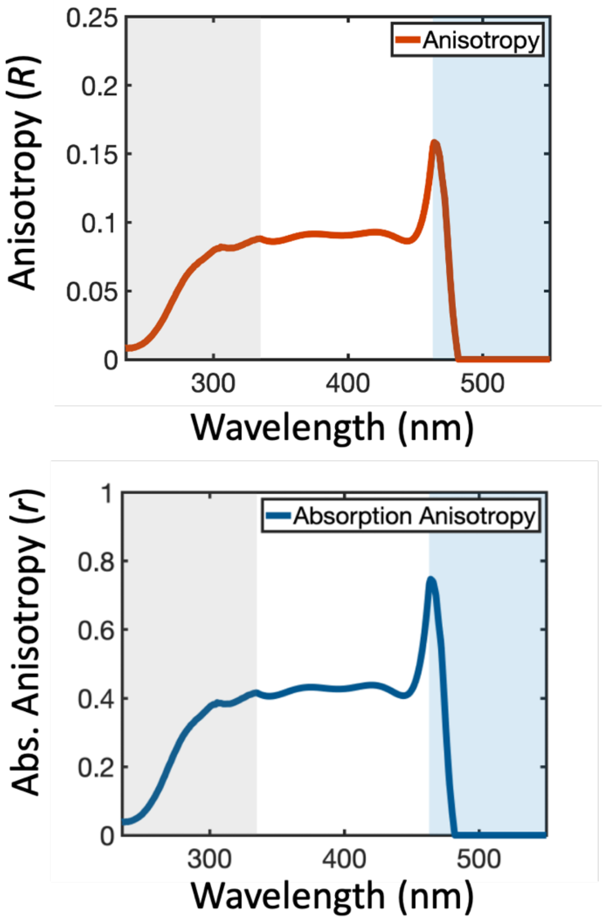

Figure S16. (top) Full anisotropy and (bottom) full absorption anisotropy of $CsPbBr_3$ nanorods. The shaded gray and blue boxes denote the extended values obtained using MG effective medium theory and by fitting PL spectra, respectively.

Figure S15 shows the fitted fluorescence anisotropy for excitation wavelengths above and below the emission peak maximum. For comparison, the fluorescence anisotropy measured for excitation wavelengths above the emission peak (values in Figure 2) is also displayed. For excitation above the emission peak, the fitted FE anisotropy values correlate well with the measured fluorescence anisotropy in Figure 2, indicating that the fitting procedure can accurately determine the anisotropy from the PL emission spectra. Interestingly, the anisotropy values monotonically decrease as the excitation shifts to wavelengths above the emission peak (i.e., data points to the right of the vertical dotted line). For wavelengths above 480 nm, we set the

anisotropy values to zero, as the absorption (and PL emission) is low at these excitation wavelengths and thus fluorescence anisotropy is not expected. This is supported by the observation in Figure S15 that the anisotropy decreases (approaching zero) with increasing excitation wavelength.

For wavelengths below 335 nm, where dielectric effects dictate the fluorescence anisotropy, we used values estimated using MG effective medium theory using bulk dielectric values. For this, we calculated the absorption coefficient parallel and perpendicular to the long axis of the nanorod using equation S9-S10 and determined the absorption anisotropy ($r = (\mu_{||} - \mu_{\perp})/(\mu_{||} + 2\,\mu_{\perp})$). Using equation 2 with q = 0.53, we then obtained the fluorescence anisotropy. Figure S16 (top figure) shows the full fluorescence anisotropy spectrum of $CsPbBr_3$ nanorods. Having determined the fluorescence anisotropy, we then obtained the full absorption anisotropy (r) of nanorod using equation 2 (Figure S16, bottom figure).

The absorption anisotropy now allows the determination of the absorption coefficient parallel and perpendicular to the long axis of the nanorod. As previously noted, the absorption anisotropy (r) is related to the absorption coefficient through ($r = (\mu_{||} - \mu_{\perp})/(\mu_{||} + 2\,\mu_{\perp})$). Defining k as the "absorption ratio" along the long and short axis ($k = \mu_{||}/\mu_{\perp}$), k can be calculated from r following the relation:

$$k = \frac{3}{(1-r)} - 2 \qquad \text{(S20)}$$

By combining equation S20 with equation S8, the average absorption coefficient spectrum (Figure 4a) can be deconvoluted into its long and short axis components. Figure 5a in the main text shows the calculated absorption coefficient spectrum for directions parallel and perpendicular to the nanorod main axis. Having determined the absorption coefficient spectra for the each axis, the iterative process can then be carried out separately to calculate the respective

complex dielectric functions. As it is discussed in the main text, for this version of the IMI calculation, the theoretical absorption coefficient spectra are calculated using equation S9 and S10. All other expressions in the calculation process are the same as previously described, except for the coefficients in the matrix inversion step, which now depend on the axis under consideration and are given by:

$$C_{j,j(\text{parallel})} = \frac{\left(\frac{1}{\sigma_e} - \frac{2\alpha_{||}^2\, \varepsilon_{I,||}^2}{\sigma_e^2}\right)}{\varepsilon_{I,||}}$$

$$C_{j,j(\text{perpendicular})} = \frac{\left(\frac{1}{\sigma_f} - \frac{2\alpha_{\perp}^2\, \varepsilon_{I,\perp}^2}{\sigma_f^2}\right)}{\varepsilon_{I,\perp}}$$

Where,

$$\sigma_e = \left(\alpha_{||}\, \varepsilon_{R,||} - n_s^2(\alpha_{||} - 1)\right)^2 + \alpha_{||}^2\, \varepsilon_{I,||}^2$$

$$\sigma_f = \left(\alpha_{\perp}\, \varepsilon_{R,\perp} - n_s^2(\alpha_{\perp} - 1)\right)^2 + \alpha_{\perp}^2\, \varepsilon_{I,\perp}^2$$

And

$$D_{j,j(\text{parallel})} = -\frac{2\alpha_{||}\left(\alpha_{||}\, \varepsilon_{R,||} - n_s^2(\alpha_{||} - 1)\right)}{\left(\alpha_{||}\, \varepsilon_{R,||} - n_s^2(\alpha_{||} - 1)\right)^2 + \alpha_{||}^2\, \varepsilon_{I,||}^2}$$

$$D_{j,j(\text{perpendicular})} = -\frac{2\alpha_{\perp}\left(\alpha_{\perp}\, \varepsilon_{R,\perp} - n_s^2(\alpha_{\perp} - 1)\right)}{\left(\alpha_{\perp}\, \varepsilon_{R,\perp} - n_s^2(\alpha_{\perp} - 1)\right)^2 + \alpha_{\perp}^2\, \varepsilon_{I,\perp}^2}$$

The resulting real and imaginary dielectric functions for directions parallel and perpendicular to the nanorod long axis (steps shown in Figure S17) are shown in the main text in Figure 5b.

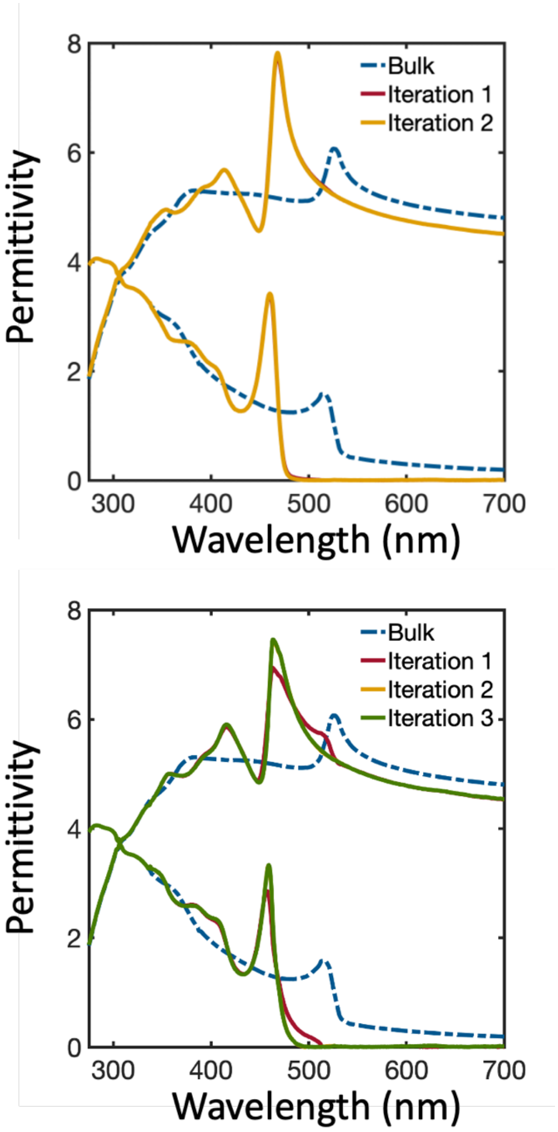

Figure S17. The real and imaginary portions of the permittivity of $CsPbBr_3$ nanorods for directions parallel (top) and perpendicular (bottom) to the nanorod long axis as found through the iterative matrix inversion process. Also shown are the bulk permittivity values for $CsPbBr_3$, which were used as the initial trial in the iterative process.

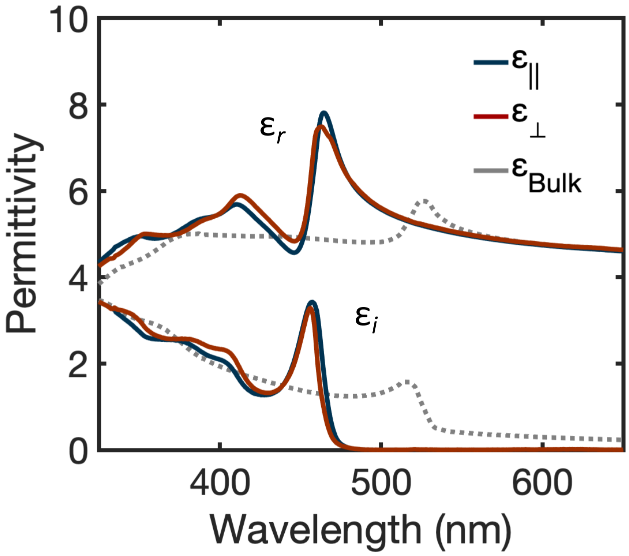

Figure S18. The real and imaginary portions of the anisotropic complex dielectric function of $CsPbBr_3$ nanorod for direction parallel (dark blue) and perpendicular (dark orange) to the nanorod long axis found after performing IMI method. The anisotropic dielectric functions of $CsPbBr_3$ nanorod are compared to the single-crystal bulk dielectric functions (dotted gray trace). Single-crystal values were extracted from Mannino *et at.*

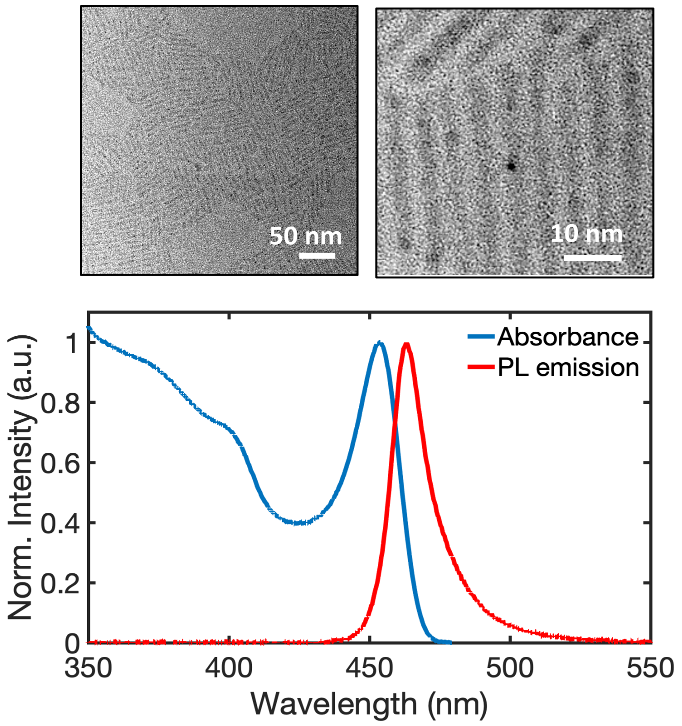

Figure S19. (top) Transmission electron micrographs and (bottom) absorption and photoluminescence emission spectra of $CsPbBr_3$ nanorods with an average length of 44.89 ± 6.34 nm and width of 4.67 ± 0.59 nm.

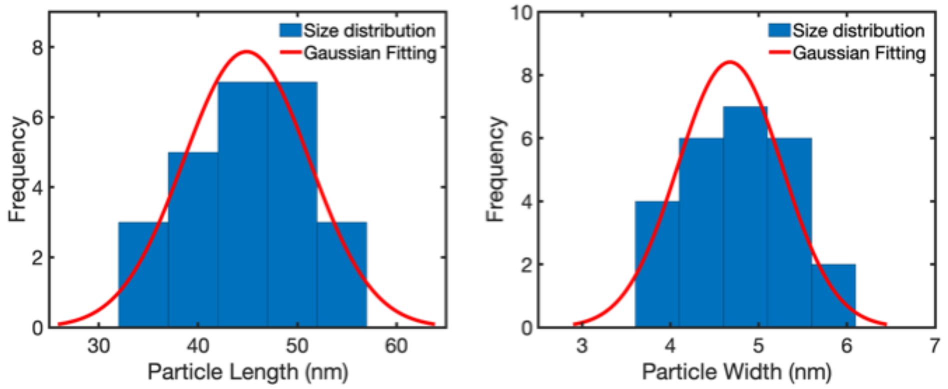

Figure S20. Distribution histograms for $CsPbBr_3$ nanorods (shown in Figure S19) (a) length and (b) width fitted with Gaussian functions (sample size = 25 particles). The average length and width were calculated to be (44.89 ± 6.34) nm and (4.67 ± 0.59) nm, respectively.

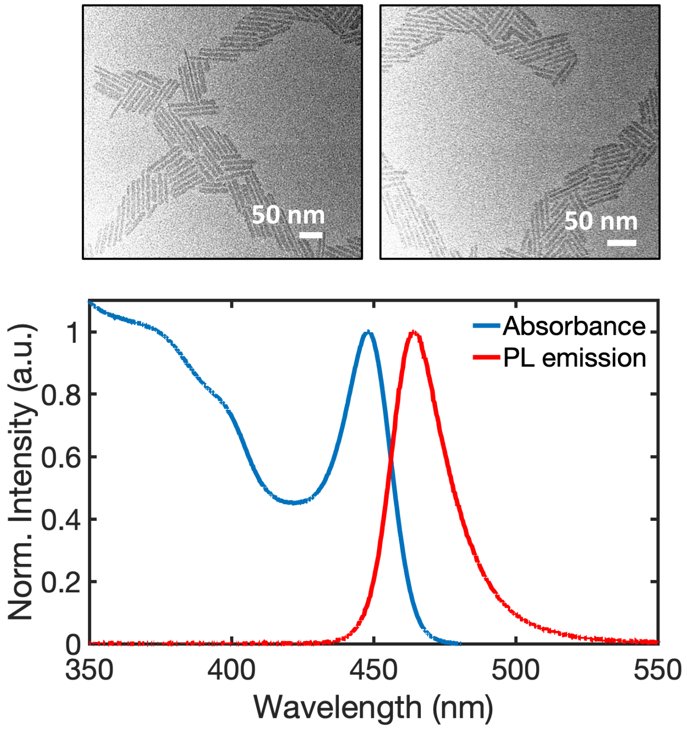

Figure S21. (top) Transmission electron micrographs and (bottom) absorption and photoluminescence emission spectra of $CsPbBr_3$ nanorods with an average length of 71.38 ± 7.65 nm and width of 7.43 ± 0.75 nm.

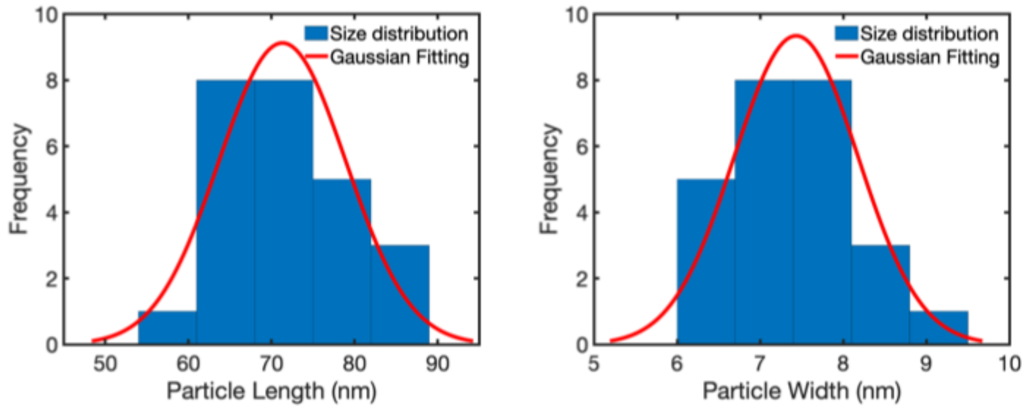

Figure S22. Distribution histograms for $CsPbBr_3$ nanorods (shown in Figure S21) (a) length and (b) width fitted with Gaussian functions (sample size = 25 particles). The average length and width were calculated to be (71.38 ± 7.65) nm and (7.43 ± 0.75) nm, respectively.

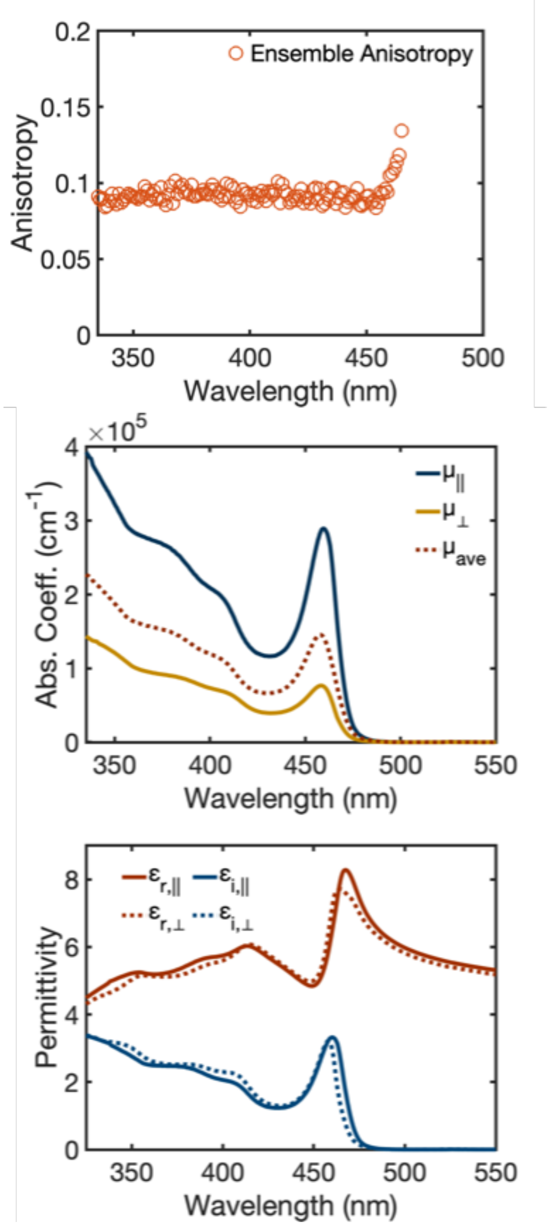

Figure S23. (Top) Ensemble fluorescence anisotropy spectra acquired for $CsPbBr_3$ nanorods in Figure S19. (Middle) Absorption coefficient spectra for directions parallel (dark blue) and perpendicular (dark yellow) to the nanorod long axis as determined from the experimental fluorescence anisotropy and MG effective medium theory. The average absorption coefficient spectrum is shown for reference (dark orange dashed curve). (b) The real and imaginary dielectric portions of $CsPbBr_3$ nanorod (shown in Figure S19) for directions parallel (solid lines) and perpendicular (dotted lines) to the nanorod long axis, respectively, found after performing the IMI method.

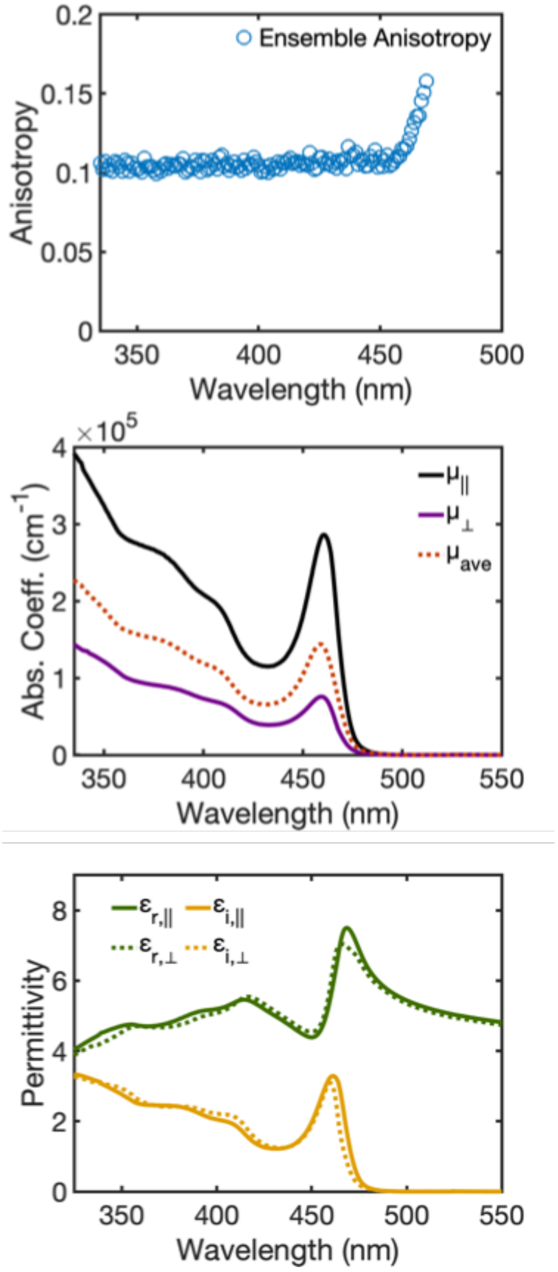

Figure S24. (Top) Ensemble fluorescence anisotropy spectra acquired for $CsPbBr_3$ nanorods in Figure S21 (Middle) Absorption coefficient spectra for directions parallel (black) and perpendicular (dark purple) to the nanorod long axis as determined from the experimental fluorescence anisotropy and MG effective medium theory. The average absorption coefficient spectrum is shown for reference (orange dashed curve). (b) The real and imaginary dielectric portions of $CsPbBr_3$ nanorod (shown in Figure S21) for directions parallel (solid lines) and perpendicular (dotted lines) to the nanorod long axis, respectively, found after performing the IMI method.

Fitting of PL emission spectra

PL emission spectra data were fitted with symmetric Voigt peaks using a custom written program in MATLAB®. Peak emission wavelengths of 463 nm and 476 nm were located from the 2D PLE map in Figure S4b, corresponding to excitonic transitions denoted as free exciton (FE) and localized exciton (LE), respectively. Scattering peaks observed in PL emission spectra scans acquired for parallel excitation and emission (i.e., I_{VV} spectra) were also fitted by Voigt line shape peaks. An additional Voigt peak was included to account for the low-energy tail emission (denoted as TE). To obtain realistic fits, the energies of the peaks were constrained within 20 meV of the peak position and peak widths at half maximum restrained between 35 and 100 meV, consistent with the observable feature widths.

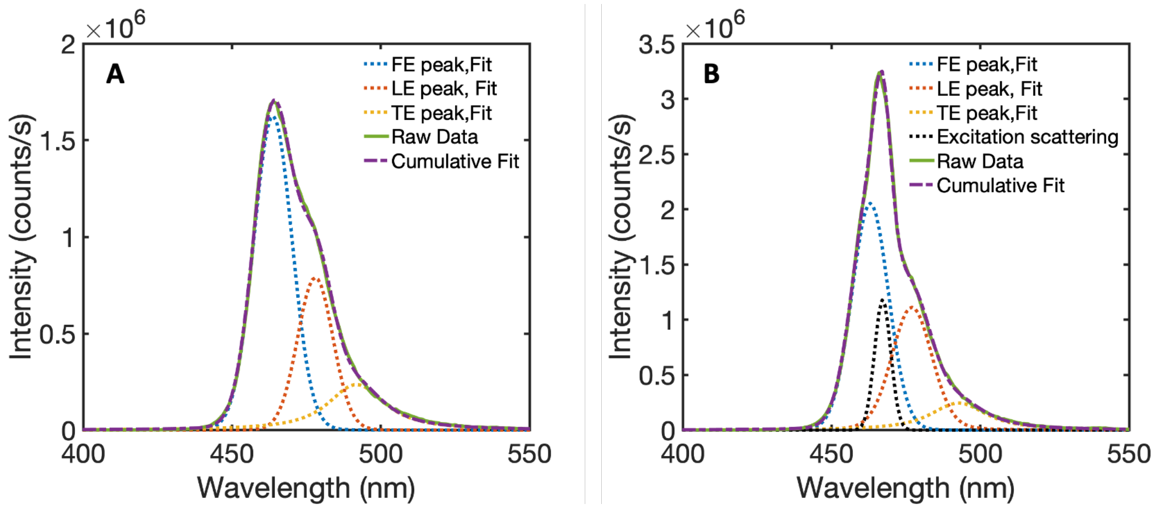

Figure S25. Examples of PL emission spectra acquired at an excitation wavelength of 466 nm for (a) polarizers in emission channel perpendicular to the polarizer in the excitation channel, and (b) with polarizers parallel in excitation and emission (I_{VV}) fitted with Voigt peaks (cumulative fit, dark purple).

Table S1. Values for real and imaginary components of the isotropic and anisotropic dielectric function of $CsPbBr_3$ nanorods found through the iterative matrix inversion method.

Wavelength (nm)	$\varepsilon_{r,\text{isotropic}}$	$\varepsilon_{I,\text{isotropic}}$	$\varepsilon_{r,\parallel}$	$\varepsilon_{r,\perp}$	$\varepsilon_{I,\parallel}$	$\varepsilon_{I,\perp}$
240.111	0.096	2.420	0.390	0.375	2.420	2.420
240.611	0.124	2.432	0.417	0.402	2.432	2.431
241.111	0.146	2.444	0.440	0.425	2.444	2.443
241.611	0.166	2.458	0.459	0.444	2.458	2.458
242.111	0.186	2.476	0.479	0.464	2.476	2.476
242.611	0.206	2.494	0.499	0.483	2.494	2.494
243.111	0.225	2.511	0.518	0.503	2.511	2.510
243.611	0.244	2.527	0.537	0.521	2.527	2.527
244.111	0.261	2.545	0.554	0.538	2.545	2.545
244.611	0.278	2.564	0.571	0.555	2.564	2.564
245.111	0.296	2.583	0.589	0.573	2.583	2.582
245.611	0.313	2.601	0.606	0.590	2.601	2.600
246.111	0.330	2.618	0.622	0.606	2.618	2.618
246.611	0.345	2.636	0.638	0.622	2.636	2.636
247.111	0.360	2.654	0.652	0.636	2.654	2.654
247.611	0.373	2.672	0.666	0.649	2.672	2.672
248.111	0.386	2.694	0.679	0.662	2.694	2.693
248.611	0.401	2.717	0.694	0.677	2.717	2.716
249.111	0.416	2.737	0.709	0.692	2.737	2.736
249.611	0.430	2.755	0.722	0.705	2.755	2.755
250.111	0.442	2.777	0.735	0.717	2.777	2.777
250.611	0.456	2.803	0.748	0.731	2.803	2.802
251.111	0.472	2.827	0.764	0.746	2.827	2.827
251.611	0.488	2.849	0.780	0.762	2.849	2.849
252.111	0.503	2.871	0.795	0.778	2.871	2.871
252.611	0.518	2.894	0.811	0.793	2.894	2.893
253.111	0.534	2.916	0.826	0.808	2.916	2.916
253.611	0.549	2.939	0.841	0.823	2.939	2.938
254.111	0.565	2.961	0.857	0.839	2.961	2.960
254.611	0.579	2.983	0.871	0.853	2.983	2.982
255.111	0.593	3.006	0.885	0.867	3.006	3.006
255.611	0.608	3.032	0.900	0.882	3.032	3.031
256.111	0.624	3.057	0.916	0.897	3.057	3.056
256.611	0.640	3.082	0.932	0.913	3.082	3.081
257.111	0.657	3.108	0.948	0.929	3.108	3.107
257.611	0.675	3.134	0.966	0.947	3.134	3.133
258.111	0.693	3.158	0.984	0.965	3.158	3.158

258.611	0.711	3.183	1.002	0.982	3.183	3.182
259.111	0.729	3.209	1.020	1.000	3.209	3.208
259.611	0.747	3.235	1.039	1.019	3.235	3.234
260.111	0.767	3.260	1.058	1.038	3.260	3.260
260.611	0.787	3.286	1.078	1.058	3.286	3.285
261.111	0.807	3.311	1.098	1.078	3.311	3.311
261.611	0.827	3.337	1.119	1.098	3.337	3.336
262.111	0.848	3.362	1.139	1.119	3.362	3.362
262.611	0.869	3.387	1.160	1.140	3.387	3.387
263.111	0.890	3.413	1.181	1.161	3.413	3.412
263.611	0.912	3.439	1.203	1.181	3.439	3.438
264.111	0.934	3.467	1.225	1.204	3.467	3.466
264.611	0.958	3.495	1.249	1.227	3.495	3.494
265.111	0.984	3.523	1.274	1.253	3.523	3.522
265.611	1.011	3.549	1.301	1.279	3.549	3.549
266.111	1.038	3.574	1.329	1.307	3.574	3.573
266.611	1.065	3.598	1.356	1.334	3.598	3.598
267.111	1.093	3.622	1.383	1.361	3.622	3.622
267.611	1.121	3.647	1.412	1.389	3.647	3.646
268.111	1.150	3.671	1.441	1.418	3.671	3.670
268.611	1.180	3.695	1.470	1.448	3.695	3.694
269.111	1.211	3.718	1.501	1.478	3.718	3.718
269.611	1.243	3.740	1.533	1.510	3.740	3.740
270.111	1.274	3.760	1.564	1.541	3.760	3.760
270.611	1.305	3.780	1.595	1.572	3.780	3.780
271.111	1.336	3.800	1.626	1.602	3.800	3.800
271.611	1.367	3.821	1.657	1.633	3.821	3.821
272.111	1.400	3.846	1.690	1.666	3.846	3.845
272.611	1.438	3.869	1.727	1.703	3.869	3.869
273.111	1.476	3.885	1.766	1.741	3.885	3.884
273.611	1.513	3.900	1.802	1.777	3.900	3.899
274.111	1.549	3.914	1.839	1.814	3.914	3.914
274.611	1.585	3.925	1.875	1.850	3.925	3.924
275.111	1.619	3.936	1.909	1.883	3.936	3.935
275.611	1.650	3.946	1.939	1.914	3.946	3.945
276.111	1.683	3.968	1.973	1.947	3.968	3.968
276.611	1.723	3.985	2.012	1.986	3.985	3.985
277.111	1.763	3.993	2.052	2.026	3.993	3.992
277.611	1.800	4.001	2.089	2.062	4.001	4.000
278.111	1.835	4.008	2.124	2.097	4.008	4.008
278.611	1.870	4.018	2.159	2.132	4.018	4.017
279.111	1.906	4.027	2.195	2.167	4.027	4.027

279.611	1.943	4.037	2.232	2.204	4.037	4.037
280.111	1.982	4.046	2.271	2.243	4.046	4.045
280.611	2.021	4.048	2.310	2.282	4.048	4.048
281.111	2.058	4.051	2.346	2.318	4.051	4.050
281.611	2.094	4.053	2.382	2.354	4.053	4.053
282.111	2.130	4.056	2.418	2.389	4.056	4.055
282.611	2.165	4.056	2.454	2.425	4.056	4.056
283.111	2.200	4.056	2.488	2.459	4.056	4.055
283.611	2.234	4.055	2.522	2.492	4.055	4.055
284.111	2.267	4.055	2.555	2.526	4.055	4.054
284.611	2.301	4.054	2.589	2.559	4.054	4.054
285.111	2.334	4.052	2.622	2.591	4.052	4.051
285.611	2.366	4.049	2.654	2.623	4.049	4.048
286.111	2.397	4.046	2.685	2.654	4.046	4.046
286.611	2.428	4.044	2.716	2.685	4.044	4.043
287.111	2.459	4.040	2.746	2.715	4.040	4.040
287.611	2.489	4.036	2.776	2.744	4.036	4.035
288.111	2.518	4.032	2.805	2.773	4.032	4.031
288.611	2.546	4.027	2.833	2.801	4.027	4.027
289.111	2.574	4.023	2.861	2.828	4.023	4.023
289.611	2.601	4.020	2.888	2.855	4.020	4.019
290.111	2.628	4.017	2.914	2.881	4.017	4.016
290.611	2.654	4.013	2.941	2.907	4.013	4.013
291.111	2.681	4.010	2.967	2.933	4.010	4.010
291.611	2.707	4.007	2.993	2.959	4.007	4.007
292.111	2.733	4.005	3.019	2.985	4.005	4.005
292.611	2.759	4.003	3.046	3.011	4.003	4.003
293.111	2.786	4.001	3.072	3.037	4.001	4.001
293.611	2.813	4.000	3.099	3.064	4.000	3.999
294.111	2.841	3.997	3.127	3.091	3.997	3.997
294.611	2.868	3.995	3.154	3.118	3.995	3.994
295.111	2.896	3.992	3.182	3.145	3.992	3.992
295.611	2.925	3.990	3.210	3.173	3.990	3.989
296.111	2.954	3.987	3.239	3.202	3.987	3.987
296.611	2.984	3.984	3.269	3.231	3.984	3.983
297.111	3.013	3.978	3.298	3.260	3.978	3.977
297.611	3.042	3.972	3.327	3.289	3.972	3.971
298.111	3.071	3.966	3.356	3.317	3.966	3.965
298.611	3.100	3.960	3.385	3.346	3.960	3.959
299.111	3.131	3.954	3.415	3.375	3.954	3.953
299.611	3.161	3.945	3.446	3.405	3.945	3.944
300.111	3.191	3.936	3.475	3.434	3.936	3.935

300.611	3.221	3.927	3.505	3.464	3.927	3.926
301.111	3.253	3.917	3.537	3.495	3.917	3.917
301.611	3.285	3.902	3.569	3.527	3.902	3.902
302.111	3.316	3.885	3.600	3.557	3.885	3.884
302.611	3.347	3.867	3.631	3.587	3.867	3.867
303.111	3.375	3.837	3.658	3.614	3.837	3.837
303.611	3.389	3.799	3.672	3.628	3.799	3.798
304.111	3.395	3.782	3.678	3.634	3.782	3.782
304.611	3.401	3.771	3.684	3.639	3.771	3.770
305.111	3.411	3.778	3.694	3.648	3.778	3.777
305.611	3.447	3.808	3.730	3.684	3.808	3.807
306.111	3.491	3.768	3.773	3.726	3.768	3.767
306.611	3.511	3.728	3.793	3.746	3.728	3.727
307.111	3.522	3.707	3.804	3.756	3.707	3.706
307.611	3.534	3.690	3.816	3.767	3.690	3.690
308.111	3.547	3.674	3.829	3.780	3.674	3.674
308.611	3.561	3.658	3.842	3.793	3.658	3.658
309.111	3.573	3.640	3.855	3.804	3.640	3.639
309.611	3.583	3.621	3.864	3.813	3.621	3.621
310.111	3.589	3.603	3.870	3.819	3.603	3.602
310.611	3.594	3.592	3.875	3.823	3.592	3.591
311.111	3.601	3.583	3.881	3.828	3.583	3.583
311.611	3.608	3.575	3.889	3.835	3.575	3.574
312.111	3.616	3.566	3.896	3.842	3.566	3.566
312.611	3.623	3.558	3.903	3.848	3.558	3.557
313.111	3.631	3.553	3.910	3.854	3.553	3.553
313.611	3.640	3.549	3.919	3.862	3.549	3.549
314.111	3.649	3.545	3.929	3.871	3.545	3.544
314.611	3.659	3.540	3.938	3.880	3.540	3.540
315.111	3.669	3.536	3.948	3.889	3.536	3.535
315.611	3.680	3.533	3.958	3.898	3.533	3.532
316.111	3.691	3.531	3.969	3.908	3.531	3.530
316.611	3.703	3.529	3.981	3.919	3.529	3.528
317.111	3.716	3.526	3.993	3.930	3.526	3.526
317.611	3.729	3.524	4.006	3.942	3.524	3.524
318.111	3.743	3.522	4.020	3.955	3.522	3.522
318.611	3.757	3.520	4.034	3.968	3.520	3.519
319.111	3.772	3.517	4.048	3.981	3.517	3.517
319.611	3.787	3.514	4.063	3.995	3.514	3.514
320.111	3.802	3.512	4.078	4.009	3.512	3.511
320.611	3.819	3.509	4.094	4.023	3.509	3.509
321.111	3.835	3.506	4.110	4.039	3.506	3.506

321.611	3.853	3.502	4.127	4.054	3.502	3.501
322.111	3.870	3.497	4.143	4.070	3.497	3.496
322.611	3.887	3.492	4.160	4.085	3.492	3.491
323.111	3.903	3.487	4.176	4.099	3.487	3.486
323.611	3.920	3.482	4.192	4.114	3.482	3.481
324.111	3.937	3.477	4.209	4.129	3.477	3.476
324.611	3.955	3.471	4.226	4.145	3.471	3.471
325.111	3.972	3.466	4.242	4.160	3.466	3.465
325.611	3.989	3.460	4.259	4.175	3.460	3.460
326.111	4.007	3.454	4.276	4.190	3.454	3.454
326.611	4.025	3.449	4.293	4.205	3.449	3.448
327.111	4.044	3.443	4.311	4.221	3.443	3.443
327.611	4.063	3.436	4.330	4.238	3.436	3.435
328.111	4.082	3.427	4.348	4.254	3.427	3.427
328.611	4.101	3.419	4.365	4.269	3.419	3.418
329.111	4.119	3.410	4.382	4.284	3.410	3.410
329.611	4.137	3.402	4.399	4.298	3.402	3.401
330.111	4.156	3.393	4.416	4.313	3.393	3.393
330.611	4.175	3.384	4.434	4.327	3.384	3.383
331.111	4.193	3.374	4.450	4.341	3.374	3.373
331.611	4.212	3.364	4.467	4.354	3.364	3.364
332.111	4.231	3.354	4.483	4.367	3.354	3.354
332.611	4.250	3.344	4.499	4.379	3.344	3.344
333.111	4.270	3.335	4.516	4.392	3.335	3.334
333.611	4.292	3.323	4.532	4.404	3.323	3.323
334.111	4.314	3.312	4.547	4.415	3.312	3.312
334.611	4.342	3.301	4.561	4.426	3.301	3.300
335.111	4.362	3.257	4.578	4.433	3.297	3.282
335.611	4.369	3.238	4.595	4.432	3.281	3.266
336.111	4.379	3.224	4.612	4.435	3.278	3.276
336.611	4.389	3.206	4.629	4.439	3.260	3.263
337.111	4.400	3.196	4.645	4.440	3.258	3.271
337.611	4.413	3.180	4.673	4.459	3.255	3.298
338.111	4.424	3.164	4.699	4.488	3.222	3.289
338.611	4.439	3.159	4.710	4.504	3.188	3.261
339.111	4.454	3.138	4.717	4.512	3.175	3.258
339.611	4.467	3.123	4.726	4.521	3.157	3.247
340.111	4.479	3.105	4.736	4.531	3.148	3.249
340.611	4.492	3.092	4.747	4.543	3.131	3.242
341.111	4.507	3.076	4.758	4.555	3.119	3.241
341.611	4.520	3.052	4.769	4.569	3.104	3.236
342.111	4.530	3.034	4.782	4.585	3.091	3.237

342.611	4.538	3.012	4.793	4.600	3.073	3.227
343.111	4.548	3.002	4.803	4.616	3.060	3.225
343.611	4.560	2.976	4.813	4.631	3.042	3.216
344.111	4.567	2.955	4.822	4.647	3.028	3.212
344.611	4.573	2.936	4.830	4.662	3.011	3.201
345.111	4.581	2.919	4.839	4.677	3.000	3.199
345.611	4.589	2.896	4.849	4.694	2.984	3.190
346.111	4.592	2.872	4.856	4.708	2.969	3.178
346.611	4.596	2.855	4.867	4.725	2.961	3.183
347.111	4.598	2.826	4.878	4.746	2.940	3.168
347.611	4.596	2.809	4.886	4.763	2.925	3.159
348.111	4.593	2.784	4.893	4.780	2.909	3.147
348.611	4.587	2.768	4.902	4.797	2.896	3.140
349.111	4.582	2.753	4.912	4.818	2.880	3.132
349.611	4.577	2.739	4.919	4.837	2.857	3.111
350.111	4.572	2.728	4.924	4.854	2.842	3.098
350.611	4.568	2.720	4.927	4.868	2.823	3.077
351.111	4.564	2.706	4.933	4.884	2.814	3.073
351.611	4.560	2.700	4.940	4.903	2.791	3.051
352.111	4.557	2.689	4.942	4.918	2.773	3.031
352.611	4.553	2.682	4.944	4.930	2.757	3.013
353.111	4.548	2.672	4.948	4.945	2.743	2.999
353.611	4.546	2.675	4.951	4.960	2.724	2.977
354.111	4.544	2.659	4.951	4.972	2.704	2.952
354.611	4.539	2.661	4.951	4.984	2.691	2.937
355.111	4.540	2.659	4.949	4.993	2.668	2.905
355.611	4.539	2.649	4.945	4.999	2.656	2.886
356.111	4.537	2.651	4.940	5.003	2.638	2.856
356.611	4.537	2.644	4.933	5.004	2.628	2.836
357.111	4.536	2.645	4.927	5.004	2.618	2.816
357.611	4.538	2.642	4.922	5.003	2.609	2.797
358.111	4.540	2.640	4.916	5.001	2.602	2.779
358.611	4.541	2.635	4.913	5.001	2.598	2.767
359.111	4.541	2.631	4.910	5.000	2.589	2.747
359.611	4.540	2.630	4.907	4.998	2.587	2.737
360.111	4.543	2.636	4.904	4.997	2.580	2.721
360.611	4.549	2.632	4.902	4.996	2.577	2.709
361.111	4.551	2.627	4.898	4.991	2.571	2.691
361.611	4.553	2.627	4.898	4.990	2.575	2.691
362.111	4.555	2.626	4.897	4.988	2.564	2.670
362.611	4.558	2.626	4.895	4.983	2.569	2.666
363.111	4.562	2.627	4.897	4.983	2.568	2.660

363.611	4.567	2.630	4.898	4.981	2.561	2.645
364.111	4.573	2.626	4.898	4.979	2.564	2.643
364.611	4.578	2.629	4.900	4.977	2.560	2.631
365.111	4.582	2.626	4.902	4.975	2.562	2.628
365.611	4.588	2.632	4.906	4.975	2.561	2.622
366.111	4.598	2.633	4.910	4.975	2.560	2.617
366.611	4.606	2.630	4.913	4.975	2.556	2.608
367.111	4.615	2.632	4.915	4.973	2.554	2.600
367.611	4.623	2.625	4.917	4.970	2.554	2.594
368.111	4.627	2.624	4.923	4.969	2.559	2.598
368.611	4.636	2.633	4.930	4.972	2.555	2.592
369.111	4.648	2.630	4.934	4.972	2.551	2.584
369.611	4.656	2.627	4.938	4.971	2.551	2.580
370.111	4.666	2.630	4.943	4.971	2.551	2.577
370.611	4.678	2.629	4.949	4.970	2.551	2.575
371.111	4.691	2.632	4.955	4.970	2.551	2.572
371.611	4.706	2.628	4.963	4.972	2.553	2.575
372.111	4.718	2.617	4.970	4.973	2.548	2.568
372.611	4.725	2.612	4.977	4.974	2.550	2.570
373.111	4.737	2.621	4.984	4.975	2.546	2.565
373.611	4.753	2.611	4.992	4.976	2.551	2.570
374.111	4.767	2.610	5.004	4.981	2.549	2.571
374.611	4.778	2.596	5.013	4.986	2.545	2.568
375.111	4.788	2.597	5.023	4.991	2.544	2.570
375.611	4.801	2.593	5.032	4.995	2.536	2.564
376.111	4.817	2.590	5.038	4.995	2.534	2.561
376.611	4.832	2.581	5.049	4.999	2.540	2.571
377.111	4.847	2.574	5.061	5.006	2.534	2.569
377.611	4.860	2.558	5.071	5.011	2.529	2.567
378.111	4.871	2.555	5.082	5.016	2.529	2.571
378.611	4.884	2.540	5.094	5.023	2.525	2.572
379.111	4.894	2.533	5.108	5.032	2.524	2.577
379.611	4.906	2.523	5.123	5.044	2.517	2.578
380.111	4.917	2.513	5.134	5.053	2.504	2.570
380.611	4.929	2.505	5.141	5.056	2.497	2.564
381.111	4.941	2.493	5.153	5.065	2.502	2.577
381.611	4.952	2.479	5.168	5.076	2.490	2.571
382.111	4.960	2.464	5.180	5.088	2.485	2.574
382.611	4.969	2.457	5.191	5.097	2.470	2.563
383.111	4.978	2.441	5.200	5.104	2.467	2.566
383.611	4.984	2.427	5.213	5.115	2.460	2.566
384.111	4.989	2.417	5.226	5.127	2.454	2.569

384.611	4.997	2.411	5.240	5.141	2.442	2.564
385.111	5.006	2.397	5.252	5.155	2.432	2.563
385.611	5.011	2.383	5.262	5.167	2.415	2.550
386.111	5.017	2.374	5.271	5.178	2.409	2.552
386.611	5.021	2.361	5.281	5.190	2.393	2.541
387.111	5.026	2.355	5.290	5.200	2.384	2.538
387.611	5.030	2.341	5.299	5.212	2.372	2.532
388.111	5.034	2.336	5.308	5.223	2.361	2.526
388.611	5.038	2.327	5.317	5.235	2.350	2.522
389.111	5.042	2.323	5.326	5.248	2.337	2.515
389.611	5.049	2.316	5.334	5.261	2.322	2.506
390.111	5.056	2.309	5.339	5.270	2.308	2.494
390.611	5.062	2.302	5.345	5.280	2.299	2.490
391.111	5.067	2.293	5.351	5.291	2.283	2.478
391.611	5.074	2.293	5.355	5.299	2.270	2.466
392.111	5.083	2.284	5.358	5.306	2.259	2.458
392.611	5.089	2.276	5.364	5.315	2.252	2.455
393.111	5.097	2.276	5.370	5.326	2.238	2.444
393.611	5.106	2.269	5.373	5.333	2.225	2.432
394.111	5.118	2.268	5.376	5.341	2.216	2.425
394.611	5.128	2.256	5.378	5.347	2.203	2.412
395.111	5.137	2.254	5.381	5.353	2.197	2.409
395.611	5.149	2.248	5.383	5.359	2.184	2.396
396.111	5.163	2.246	5.385	5.364	2.179	2.392
396.611	5.179	2.237	5.388	5.369	2.170	2.383
397.111	5.193	2.228	5.391	5.375	2.165	2.381
397.611	5.209	2.222	5.396	5.382	2.159	2.376
398.111	5.226	2.210	5.401	5.390	2.151	2.371
398.611	5.241	2.197	5.406	5.398	2.144	2.366
399.111	5.258	2.189	5.410	5.403	2.135	2.357
399.611	5.276	2.174	5.415	5.411	2.134	2.359
400.111	5.294	2.160	5.422	5.421	2.125	2.352
400.611	5.312	2.141	5.427	5.427	2.117	2.345
401.111	5.329	2.125	5.433	5.435	2.116	2.346
401.611	5.348	2.104	5.441	5.445	2.109	2.341
402.111	5.365	2.080	5.450	5.457	2.106	2.343
402.611	5.381	2.056	5.459	5.468	2.094	2.333
403.111	5.394	2.025	5.466	5.478	2.091	2.333
403.611	5.405	2.002	5.476	5.490	2.084	2.330
404.111	5.417	1.972	5.488	5.504	2.080	2.331
404.611	5.428	1.944	5.501	5.521	2.070	2.327
405.111	5.437	1.908	5.513	5.537	2.060	2.321

405.611	5.441	1.872	5.526	5.554	2.053	2.319
406.111	5.441	1.840	5.539	5.573	2.039	2.311
406.611	5.441	1.808	5.551	5.591	2.026	2.301
407.111	5.440	1.778	5.564	5.610	2.017	2.298
407.611	5.436	1.741	5.578	5.631	2.002	2.288
408.111	5.428	1.709	5.592	5.653	1.986	2.278
408.611	5.420	1.682	5.606	5.674	1.967	2.263
409.111	5.410	1.650	5.619	5.697	1.951	2.252
409.611	5.397	1.621	5.632	5.721	1.930	2.235
410.111	5.385	1.600	5.644	5.743	1.907	2.215
410.611	5.372	1.570	5.655	5.766	1.884	2.195
411.111	5.354	1.545	5.664	5.786	1.856	2.165
411.611	5.338	1.527	5.671	5.805	1.834	2.145
412.111	5.323	1.506	5.678	5.824	1.807	2.116
412.611	5.306	1.482	5.684	5.843	1.781	2.089
413.111	5.287	1.467	5.688	5.860	1.749	2.054
413.611	5.269	1.451	5.687	5.872	1.717	2.016
414.111	5.252	1.435	5.684	5.880	1.689	1.981
414.611	5.234	1.418	5.681	5.887	1.662	1.948
415.111	5.215	1.405	5.677	5.893	1.635	1.915
415.611	5.195	1.389	5.670	5.896	1.603	1.874
416.111	5.175	1.383	5.659	5.894	1.576	1.837
416.611	5.155	1.365	5.649	5.891	1.554	1.806
417.111	5.133	1.363	5.638	5.887	1.527	1.769
417.611	5.116	1.357	5.623	5.878	1.503	1.734
418.111	5.099	1.351	5.610	5.870	1.486	1.708
418.611	5.080	1.339	5.596	5.861	1.461	1.673
419.111	5.061	1.337	5.579	5.847	1.440	1.642
419.611	5.043	1.331	5.562	5.833	1.427	1.619
420.111	5.026	1.327	5.547	5.821	1.409	1.593
420.611	5.007	1.318	5.530	5.805	1.390	1.564
421.111	4.986	1.319	5.511	5.787	1.378	1.543
421.611	4.969	1.317	5.494	5.771	1.366	1.523
422.111	4.952	1.316	5.477	5.755	1.353	1.502
422.611	4.934	1.312	5.460	5.737	1.340	1.481
423.111	4.914	1.312	5.442	5.720	1.330	1.464
423.611	4.897	1.313	5.422	5.700	1.317	1.442
424.111	4.878	1.312	5.404	5.681	1.314	1.433
424.611	4.860	1.315	5.384	5.660	1.299	1.410
425.111	4.842	1.315	5.364	5.638	1.300	1.404
425.611	4.823	1.318	5.348	5.620	1.296	1.395
426.111	4.803	1.317	5.332	5.603	1.291	1.386

426.611	4.782	1.322	5.315	5.584	1.282	1.371
427.111	4.762	1.329	5.296	5.564	1.282	1.366
427.611	4.743	1.337	5.280	5.546	1.278	1.358
428.111	4.724	1.343	5.264	5.528	1.276	1.351
428.611	4.704	1.350	5.245	5.508	1.269	1.339
429.111	4.684	1.359	5.227	5.488	1.272	1.338
429.611	4.666	1.370	5.210	5.469	1.271	1.333
430.111	4.646	1.379	5.194	5.452	1.272	1.331
430.611	4.626	1.393	5.177	5.433	1.269	1.324
431.111	4.606	1.400	5.159	5.414	1.271	1.323
431.611	4.583	1.417	5.142	5.396	1.272	1.322
432.111	4.563	1.434	5.124	5.377	1.273	1.319
432.611	4.542	1.450	5.107	5.358	1.276	1.320
433.111	4.521	1.473	5.089	5.340	1.278	1.319
433.611	4.503	1.496	5.072	5.322	1.282	1.321
434.111	4.484	1.517	5.052	5.302	1.283	1.319
434.611	4.465	1.541	5.032	5.281	1.289	1.322
435.111	4.446	1.565	5.013	5.260	1.297	1.328
435.611	4.428	1.595	4.995	5.242	1.306	1.335
436.111	4.410	1.624	4.977	5.223	1.312	1.339
436.611	4.393	1.657	4.957	5.204	1.321	1.345
437.111	4.378	1.692	4.938	5.184	1.331	1.353
437.611	4.363	1.725	4.920	5.166	1.343	1.363
438.111	4.347	1.764	4.900	5.147	1.352	1.370
438.611	4.333	1.806	4.881	5.129	1.367	1.384
439.111	4.320	1.849	4.861	5.109	1.376	1.390
439.611	4.308	1.899	4.839	5.088	1.394	1.405
440.111	4.301	1.953	4.819	5.068	1.413	1.421
440.611	4.295	2.000	4.799	5.048	1.430	1.435
441.111	4.290	2.062	4.779	5.029	1.454	1.456
441.611	4.291	2.125	4.761	5.012	1.479	1.478
442.111	4.293	2.185	4.743	4.995	1.500	1.497
442.611	4.301	2.258	4.725	4.978	1.525	1.519
443.111	4.314	2.325	4.706	4.961	1.551	1.542
443.611	4.330	2.401	4.688	4.944	1.583	1.570
444.111	4.354	2.477	4.672	4.929	1.613	1.596
444.611	4.383	2.556	4.655	4.913	1.648	1.627
445.111	4.421	2.640	4.641	4.901	1.684	1.661
445.611	4.463	2.716	4.627	4.888	1.719	1.690
446.111	4.515	2.809	4.612	4.875	1.760	1.727
446.611	4.578	2.887	4.600	4.864	1.804	1.766
447.111	4.648	2.971	4.588	4.854	1.849	1.804

447.611	4.728	3.052	4.579	4.844	1.901	1.850
448.111	4.818	3.131	4.574	4.840	1.956	1.901
448.611	4.920	3.209	4.570	4.836	2.006	1.943
449.111	5.033	3.271	4.568	4.833	2.069	2.000
449.611	5.154	3.335	4.572	4.836	2.132	2.057
450.111	5.287	3.384	4.578	4.841	2.193	2.112
450.611	5.430	3.425	4.589	4.849	2.266	2.179
451.111	5.582	3.447	4.605	4.864	2.332	2.241
451.611	5.736	3.448	4.626	4.882	2.407	2.310
452.111	5.893	3.443	4.653	4.906	2.481	2.381
452.611	6.051	3.412	4.686	4.936	2.557	2.453
453.111	6.206	3.371	4.726	4.974	2.636	2.533
453.611	6.361	3.319	4.771	5.017	2.709	2.602
454.111	6.512	3.238	4.824	5.070	2.795	2.691
454.611	6.650	3.143	4.887	5.133	2.868	2.765
455.111	6.781	3.045	4.955	5.205	2.945	2.847
455.611	6.903	2.926	5.032	5.287	3.020	2.923
456.111	7.011	2.799	5.117	5.380	3.092	3.000
456.611	7.106	2.665	5.213	5.488	3.164	3.076
457.111	7.189	2.524	5.316	5.609	3.221	3.138
457.611	7.259	2.379	5.428	5.742	3.283	3.197
458.111	7.315	2.227	5.549	5.890	3.331	3.243
458.611	7.356	2.075	5.678	6.052	3.376	3.275
459.111	7.382	1.923	5.816	6.228	3.403	3.287
459.611	7.397	1.780	5.956	6.410	3.418	3.262
460.111	7.401	1.633	6.101	6.596	3.429	3.224
460.611	7.392	1.489	6.250	6.782	3.421	3.140
461.111	7.372	1.359	6.401	6.957	3.407	3.031
461.611	7.344	1.229	6.559	7.119	3.386	2.887
462.111	7.308	1.111	6.720	7.256	3.340	2.701
462.611	7.265	0.995	6.882	7.342	3.287	2.469
463.111	7.217	0.893	7.055	7.400	3.220	2.244
463.611	7.163	0.791	7.223	7.430	3.098	2.000
464.111	7.104	0.704	7.373	7.450	2.963	1.819
464.611	7.042	0.621	7.503	7.475	2.799	1.662
465.111	6.977	0.550	7.610	7.480	2.629	1.480
465.611	6.913	0.486	7.695	7.485	2.447	1.358
466.111	6.848	0.429	7.756	7.488	2.262	1.240
466.611	6.783	0.377	7.794	7.480	2.076	1.133
467.111	6.719	0.333	7.809	7.464	1.897	1.027
467.611	6.656	0.296	7.811	7.430	1.740	0.930
468.111	6.595	0.266	7.804	7.400	1.582	0.836

468.611	6.539	0.239	7.783	7.370	1.428	0.752
469.111	6.485	0.214	7.752	7.340	1.290	0.669
469.611	6.433	0.192	7.712	7.310	1.155	0.597
470.111	6.381	0.172	7.665	7.280	1.034	0.529
470.611	6.333	0.161	7.612	7.260	0.918	0.471
471.111	6.288	0.144	7.554	7.240	0.816	0.418
471.611	6.246	0.137	7.492	7.215	0.715	0.370
472.111	6.206	0.120	7.425	7.180	0.631	0.327
472.611	6.166	0.113	7.357	7.140	0.552	0.290
473.111	6.130	0.104	7.288	7.097	0.484	0.259
473.611	6.093	0.093	7.220	7.050	0.424	0.234
474.111	6.058	0.090	7.152	7.002	0.370	0.211
474.611	6.026	0.084	7.085	6.952	0.321	0.190
475.111	5.995	0.077	7.018	6.902	0.279	0.171
475.611	5.965	0.070	6.953	6.852	0.245	0.154
476.111	5.936	0.068	6.891	6.803	0.218	0.144
476.611	5.909	0.060	6.833	6.757	0.193	0.129
477.111	5.883	0.057	6.779	6.712	0.171	0.124
477.611	5.858	0.051	6.725	6.667	0.150	0.109
478.111	5.832	0.045	6.673	6.624	0.133	0.102
478.611	5.807	0.041	6.625	6.584	0.123	0.094
479.111	5.782	0.037	6.579	6.544	0.109	0.084
479.611	5.758	0.035	6.537	6.508	0.102	0.082
480.111	5.736	0.033	6.497	6.473	0.088	0.076
480.611	5.714	0.029	6.458	6.437	0.081	0.070
481.111	5.692	0.027	6.421	6.404	0.072	0.064
481.611	5.671	0.023	6.384	6.371	0.063	0.063
482.111	5.650	0.023	6.349	6.340	0.059	0.056
482.611	5.631	0.022	6.316	6.313	0.054	0.053
483.111	5.613	0.020	6.285	6.287	0.048	0.047
483.611	5.595	0.017	6.254	6.260	0.042	0.042
484.111	5.577	0.017	6.225	6.236	0.040	0.038
484.611	5.560	0.014	6.198	6.212	0.034	0.034
485.111	5.543	0.012	6.170	6.191	0.029	0.033
485.611	5.525	0.010	6.142	6.172	0.026	0.030
486.111	5.508	0.009	6.116	6.149	0.023	0.027
486.611	5.492	0.008	6.091	6.126	0.021	0.026
487.111	5.476	0.006	6.066	6.103	0.019	0.021
487.611	5.460	0.006	6.043	6.081	0.018	0.022
488.111	5.445	0.005	6.021	6.060	0.017	0.020
488.611	5.429	0.005	5.999	6.039	0.015	0.019
489.111	5.415	0.007	5.978	6.019	0.014	0.016

489.611	5.403	0.007	5.958	5.999	0.012	0.016
490.111	5.390	0.004	5.938	5.978	0.012	0.013
490.611	5.376	0.004	5.919	5.960	0.011	0.011
491.111	5.363	0.003	5.901	5.943	0.011	0.009
491.611	5.350	0.002	5.883	5.925	0.009	0.009
492.111	5.336	0.000	5.866	5.908	0.009	0.007
492.611	5.323	0.002	5.849	5.892	0.007	0.005
493.111	5.312	0.003	5.833	5.875	0.006	0.006
493.611	5.300	0.003	5.816	5.857	0.005	0.005
494.111	5.289	0.004	5.800	5.840	0.005	0.005
494.611	5.279	0.002	5.785	5.825	0.004	0.006
495.111	5.267	0.000	5.770	5.808	0.003	0.007
495.611	5.256	0.002	5.755	5.792	0.003	0.004
496.111	5.246	0.002	5.740	5.776	0.003	0.004
496.611	5.235	0.001	5.726	5.761	0.003	0.003
497.111	5.224	0.001	5.712	5.746	0.003	0.001
497.611	5.214	0.002	5.699	5.735	0.004	0.000
498.111	5.205	0.000	5.687	5.723	0.002	0.002
498.611	5.194	0.000	5.674	5.709	0.002	0.002
499.111	5.184	0.000	5.661	5.696	0.002	0.002
499.611	5.174	0.001	5.649	5.683	0.001	0.004
500.111	5.165	0.002	5.636	5.669	0.000	0.002
500.611	5.157	0.002	5.624	5.655	0.001	0.000
501.111	5.148	0.001	5.613	5.644	0.001	0.002
501.611	5.139	0.002	5.601	5.632	0.001	0.002
502.111	5.130	0.003	5.591	5.622	0.002	0.001
502.611	5.122	0.001	5.580	5.613	0.001	0.001
503.111	5.113	0.000	5.569	5.600	0.000	0.002
503.611	5.105	0.002	5.558	5.589	0.001	0.000
504.111	5.097	0.001	5.548	5.580	0.001	0.000
504.611	5.088	0.000	5.538	5.570	0.000	0.000
505.111	5.079	0.001	5.528	5.558	0.000	0.001
505.611	5.071	0.003	5.518	5.549	0.001	0.002
506.111	5.064	0.004	5.509	5.539	0.000	0.002
506.611	5.058	0.003	5.499	5.528	0.000	0.001
507.111	5.051	0.004	5.490	5.518	0.000	0.002
507.611	5.044	0.002	5.480	5.508	0.001	0.002
508.111	5.036	0.001	5.471	5.499	0.001	0.001
508.611	5.029	0.001	5.463	5.491	0.001	0.000
509.111	5.021	0.001	5.454	5.482	0.001	0.002
509.611	5.014	0.002	5.445	5.473	0.001	0.001
510.111	5.007	0.002	5.437	5.466	0.001	0.000

510.611	5.000	0.002	5.429	5.458	0.001	0.001
511.111	4.993	0.002	5.421	5.449	0.000	0.002
511.611	4.987	0.003	5.413	5.440	0.001	0.004
512.111	4.980	0.001	5.405	5.433	0.000	0.003
512.611	4.972	0.002	5.397	5.424	0.000	0.004
513.111	4.966	0.003	5.388	5.414	0.000	0.002
513.611	4.959	0.002	5.381	5.406	0.001	0.001
514.111	4.952	0.003	5.374	5.400	0.002	0.001
514.611	4.945	0.004	5.367	5.394	0.002	0.001
515.111	4.939	0.005	5.360	5.388	0.002	0.002
515.611	4.934	0.006	5.353	5.382	0.001	0.002
516.111	4.927	0.006	5.346	5.374	0.001	0.002
516.611	4.922	0.009	5.338	5.365	0.000	0.002
517.111	4.916	0.009	5.331	5.357	0.001	0.003
517.611	4.912	0.012	5.324	5.350	0.001	0.001
518.111	4.908	0.013	5.317	5.344	0.001	0.002
518.611	4.905	0.012	5.311	5.337	0.001	0.003
519.111	4.901	0.010	5.304	5.330	0.001	0.002
519.611	4.895	0.009	5.298	5.324	0.002	0.003
520.111	4.890	0.008	5.291	5.317	0.000	0.004
520.611	4.885	0.009	5.284	5.309	0.001	0.005
521.111	4.880	0.008	5.278	5.303	0.002	0.006
521.611	4.874	0.006	5.272	5.296	0.001	0.006
522.111	4.868	0.007	5.265	5.288	0.002	0.008
522.611	4.864	0.009	5.259	5.282	0.002	0.008
523.111	4.859	0.006	5.253	5.275	0.003	0.011
523.611	4.854	0.006	5.247	5.270	0.003	0.012
524.111	4.848	0.007	5.241	5.264	0.004	0.012
524.611	4.843	0.007	5.236	5.259	0.005	0.009
525.111	4.840	0.011	5.230	5.254	0.005	0.008
525.611	4.836	0.006	5.225	5.249	0.006	0.008
526.111	4.832	0.007	5.221	5.247	0.007	0.009
526.611	4.827	0.004	5.216	5.245	0.007	0.007
527.111	4.821	0.004	5.212	5.242	0.005	0.005
527.611	4.815	0.005	5.206	5.236	0.005	0.006
528.111	4.811	0.007	5.201	5.231	0.004	0.008
528.611	4.807	0.006	5.196	5.226	0.005	0.006
529.111	4.804	0.008	5.191	5.222	0.004	0.006
529.611	4.800	0.004	5.186	5.216	0.003	0.007
530.111	4.795	0.004	5.180	5.210	0.004	0.006
530.611	4.790	0.005	5.175	5.206	0.005	0.010
531.111	4.787	0.005	5.171	5.202	0.003	0.006

531.611	4.782	0.003	5.166	5.196	0.003	0.007
532.111	4.778	0.004	5.161	5.191	0.004	0.003
532.611	4.773	0.001	5.155	5.185	0.004	0.003
533.111	4.768	0.003	5.152	5.183	0.006	0.004
533.611	4.765	0.002	5.148	5.180	0.003	0.007
534.111	4.760	0.001	5.143	5.176	0.004	0.005
534.611	4.755	0.000	5.138	5.171	0.002	0.008
535.111	4.751	0.002	5.133	5.164	0.002	0.004
535.611	4.747	0.000	5.128	5.158	0.003	0.004
536.111	4.742	0.001	5.123	5.154	0.004	0.005
536.611	4.738	0.002	5.119	5.151	0.003	0.004
537.111	4.733	0.001	5.116	5.148	0.005	0.003
537.611	4.730	0.004	5.112	5.145	0.002	0.004
538.111	4.726	0.002	5.107	5.139	0.002	0.001
538.611	4.722	0.002	5.102	5.135	0.003	0.003
539.111	4.719	0.004	5.098	5.131	0.003	0.002
539.611	4.716	0.002	5.094	5.127	0.002	0.001
540.111	4.711	0.001	5.090	5.123	0.002	0.000
540.611	4.707	0.002	5.086	5.118	0.000	0.002
541.111	4.704	0.001	5.081	5.113	0.002	0.000
541.611	4.700	0.001	5.077	5.109	0.001	0.001
542.111	4.695	0.001	5.073	5.105	0.001	0.002
542.611	4.691	0.001	5.068	5.099	0.000	0.001
543.111	4.687	0.001	5.064	5.095	0.001	0.004
543.611	4.684	0.002	5.060	5.091	0.000	0.002
544.111	4.680	0.003	5.056	5.085	0.001	0.002
544.611	4.677	0.005	5.052	5.081	0.001	0.003
545.111	4.675	0.004	5.048	5.077	0.001	0.002
545.611	4.672	0.004	5.044	5.073	0.002	0.001
546.111	4.668	0.001	5.041	5.070	0.001	0.002
546.611	4.664	0.003	5.037	5.066	0.001	0.001
547.111	4.662	0.004	5.033	5.063	0.002	0.001
547.611	4.659	0.001	5.030	5.060	0.001	0.001
548.111	4.655	0.000	5.026	5.055	0.000	0.001
548.611	4.651	0.001	5.022	5.051	0.001	0.001
549.111	4.648	0.002	5.019	5.048	0.001	0.002
549.611	4.644	0.000	5.015	5.044	0.000	0.002
550.111	4.641	0.002	5.011	5.039	0.001	0.005
550.611	4.638	0.000	5.007	5.035	0.001	0.004
551.111	4.634	0.001	5.003	5.031	0.001	0.004
551.611	4.630	0.001	4.999	5.027	0.001	0.001
552.111	4.628	0.003	4.996	5.023	0.001	0.003

552.611	4.625	0.000	4.993	5.021	0.003	0.004
553.111	4.620	0.002	4.990	5.019	0.002	0.001
553.611	4.618	0.004	4.987	5.017	0.002	0.000
554.111	4.616	0.002	4.984	5.013	0.001	0.001
554.611	4.614	0.004	4.980	5.008	0.002	0.002
555.111	4.611	0.001	4.977	5.007	0.002	0.000
555.611	4.607	0.000	4.974	5.004	0.001	0.002
556.111	4.603	0.001	4.970	4.999	0.000	0.000
556.611	4.601	0.001	4.967	4.995	0.001	0.001
557.111	4.597	0.000	4.964	4.992	0.001	0.001
557.611	4.593	0.001	4.961	4.989	0.000	0.003
558.111	4.591	0.002	4.957	4.985	0.001	0.000
558.611	4.588	0.002	4.954	4.982	0.000	0.002
559.111	4.585	0.001	4.951	4.978	0.001	0.004
559.611	4.582	0.002	4.947	4.974	0.001	0.002
560.111	4.579	0.002	4.945	4.972	0.002	0.004
560.611	4.577	0.002	4.941	4.968	0.000	0.001
561.111	4.574	0.003	4.938	4.964	0.001	0.000
561.611	4.571	0.001	4.935	4.962	0.002	0.001
562.111	4.567	0.002	4.933	4.960	0.001	0.001
562.611	4.564	0.002	4.930	4.958	0.002	0.000
563.111	4.562	0.004	4.927	4.956	0.000	0.001
563.611	4.560	0.002	4.924	4.951	0.000	0.002
564.111	4.558	0.004	4.921	4.947	0.001	0.002
564.611	4.555	0.001	4.918	4.945	0.001	0.001
565.111	4.551	0.002	4.915	4.941	0.000	0.002
565.611	4.548	0.004	4.911	4.937	0.001	0.002
566.111	4.546	0.004	4.909	4.934	0.001	0.002
566.611	4.545	0.005	4.906	4.932	0.001	0.002
567.111	4.542	0.002	4.903	4.929	0.001	0.001
567.611	4.539	0.004	4.900	4.925	0.001	0.002
568.111	4.537	0.004	4.897	4.923	0.001	0.002
568.611	4.534	0.002	4.895	4.920	0.001	0.004
569.111	4.530	0.004	4.892	4.918	0.001	0.002
569.611	4.529	0.006	4.889	4.914	0.000	0.003
570.111	4.528	0.005	4.886	4.911	0.001	0.001
570.611	4.525	0.003	4.883	4.908	0.001	0.002
571.111	4.522	0.003	4.881	4.906	0.002	0.004
571.611	4.519	0.001	4.878	4.903	0.001	0.004
572.111	4.515	0.003	4.876	4.901	0.002	0.005
572.611	4.513	0.004	4.873	4.898	0.001	0.002
573.111	4.512	0.006	4.870	4.893	0.001	0.003

573.611	4.509	0.001	4.867	4.891	0.003	0.004
574.111	4.506	0.005	4.865	4.889	0.002	0.002
574.611	4.503	0.004	4.863	4.888	0.003	0.004
575.111	4.501	0.005	4.861	4.886	0.001	0.006
575.611	4.499	0.004	4.858	4.882	0.002	0.004
576.111	4.496	0.004	4.856	4.880	0.002	0.003
576.611	4.493	0.006	4.853	4.877	0.001	0.003
577.111	4.492	0.006	4.850	4.873	0.002	0.001
577.611	4.489	0.004	4.848	4.872	0.004	0.003
578.111	4.486	0.005	4.846	4.872	0.003	0.004
578.611	4.483	0.005	4.844	4.869	0.002	0.005
579.111	4.481	0.010	4.841	4.866	0.002	0.001
579.611	4.481	0.008	4.838	4.862	0.001	0.004
580.111	4.479	0.009	4.835	4.858	0.002	0.004
580.611	4.477	0.007	4.833	4.856	0.002	0.004
581.111	4.474	0.009	4.831	4.855	0.003	0.004
581.611	4.473	0.010	4.829	4.852	0.001	0.003
582.111	4.471	0.008	4.826	4.848	0.003	0.005
582.611	4.469	0.009	4.824	4.846	0.002	0.006
583.111	4.468	0.009	4.821	4.844	0.003	0.004
583.611	4.466	0.007	4.819	4.842	0.003	0.005
584.111	4.462	0.007	4.816	4.838	0.002	0.004
584.611	4.460	0.011	4.814	4.836	0.004	0.009
585.111	4.459	0.010	4.812	4.835	0.004	0.008
585.611	4.457	0.009	4.810	4.832	0.003	0.008
586.111	4.455	0.009	4.807	4.829	0.003	0.006
586.611	4.452	0.012	4.804	4.825	0.003	0.008
587.111	4.452	0.013	4.803	4.823	0.006	0.009
587.611	4.452	0.011	4.801	4.823	0.005	0.007
588.111	4.450	0.009	4.800	4.822	0.005	0.008
588.611	4.448	0.010	4.797	4.820	0.004	0.008
589.111	4.447	0.010	4.795	4.817	0.005	0.006
589.611	4.445	0.008	4.793	4.816	0.006	0.006
590.111	4.442	0.007	4.791	4.814	0.005	0.010
590.611	4.440	0.008	4.789	4.812	0.005	0.008
591.111	4.437	0.007	4.788	4.812	0.005	0.008
591.611	4.435	0.008	4.786	4.809	0.004	0.008
592.111	4.434	0.008	4.783	4.805	0.004	0.010
592.611	4.431	0.005	4.781	4.803	0.007	0.012
593.111	4.428	0.008	4.779	4.803	0.006	0.010
593.611	4.426	0.008	4.777	4.801	0.005	0.008
594.111	4.424	0.008	4.775	4.798	0.005	0.009

594.611	4.421	0.009	4.773	4.796	0.007	0.009
595.111	4.420	0.012	4.772	4.797	0.008	0.007
595.611	4.421	0.013	4.771	4.797	0.006	0.006
596.111	4.419	0.009	4.769	4.795	0.005	0.007
596.611	4.417	0.010	4.767	4.793	0.006	0.006
597.111	4.415	0.008	4.766	4.792	0.006	0.007
597.611	4.413	0.010	4.764	4.790	0.004	0.007
598.111	4.411	0.010	4.761	4.787	0.004	0.004
598.611	4.409	0.008	4.759	4.784	0.005	0.007
599.111	4.407	0.009	4.757	4.782	0.004	0.007
599.611	4.405	0.010	4.755	4.780	0.005	0.007
600.111	4.402	0.010	4.754	4.779	0.005	0.008
600.611	4.401	0.013	4.751	4.776	0.003	0.011
601.111	4.401	0.011	4.748	4.772	0.004	0.011
601.611	4.398	0.010	4.747	4.770	0.005	0.008
602.111	4.397	0.013	4.745	4.767	0.005	0.009
602.611	4.396	0.010	4.742	4.765	0.005	0.007
603.111	4.393	0.011	4.741	4.764	0.007	0.009
603.611	4.392	0.012	4.740	4.765	0.007	0.008
604.111	4.390	0.011	4.739	4.764	0.005	0.007
604.611	4.389	0.014	4.737	4.762	0.006	0.008
605.111	4.387	0.011	4.735	4.759	0.004	0.008
605.611	4.386	0.014	4.733	4.757	0.006	0.008
606.111	4.385	0.013	4.731	4.756	0.006	0.011
606.611	4.384	0.012	4.729	4.753	0.005	0.010
607.111	4.382	0.011	4.727	4.750	0.005	0.009
607.611	4.379	0.010	4.725	4.748	0.006	0.011
608.111	4.376	0.013	4.723	4.746	0.006	0.009
608.611	4.375	0.014	4.722	4.745	0.008	0.010
609.111	4.375	0.015	4.721	4.745	0.006	0.011
609.611	4.373	0.013	4.719	4.742	0.006	0.010
610.111	4.371	0.017	4.717	4.741	0.008	0.012
610.611	4.372	0.018	4.716	4.740	0.006	0.009
611.111	4.371	0.014	4.714	4.737	0.007	0.012
611.611	4.370	0.016	4.712	4.736	0.007	0.012
612.111	4.368	0.013	4.711	4.734	0.007	0.010
612.611	4.366	0.014	4.709	4.733	0.008	0.010
613.111	4.365	0.015	4.708	4.732	0.006	0.009
613.611	4.362	0.013	4.706	4.730	0.008	0.011
614.111	4.361	0.018	4.705	4.730	0.008	0.012
614.611	4.362	0.017	4.704	4.729	0.007	0.013
615.111	4.360	0.014	4.702	4.727	0.007	0.011

615.611	4.358	0.016	4.699	4.723	0.006	0.015
616.111	4.358	0.018	4.697	4.720	0.008	0.016
616.611	4.357	0.016	4.696	4.719	0.008	0.012
617.111	4.356	0.016	4.695	4.719	0.009	0.014
617.611	4.355	0.017	4.693	4.717	0.008	0.011
618.111	4.353	0.013	4.692	4.716	0.010	0.012
618.611	4.351	0.016	4.692	4.718	0.011	0.013
619.111	4.351	0.016	4.691	4.717	0.008	0.011
619.611	4.350	0.017	4.689	4.716	0.010	0.016
620.111	4.349	0.014	4.688	4.715	0.007	0.015
620.611	4.347	0.016	4.685	4.711	0.008	0.012
621.111	4.347	0.017	4.684	4.710	0.009	0.014
621.611	4.346	0.015	4.682	4.708	0.007	0.015
622.111	4.345	0.019	4.681	4.706	0.011	0.014
622.611	4.345	0.015	4.681	4.708	0.010	0.014
623.111	4.344	0.016	4.679	4.706	0.008	0.014
623.611	4.343	0.014	4.677	4.704	0.009	0.012
624.111	4.342	0.013	4.676	4.704	0.010	0.014
624.611	4.341	0.014	4.675	4.704	0.009	0.013
625.111	4.340	0.013	4.674	4.703	0.010	0.015
625.611	4.339	0.010	4.673	4.703	0.010	0.012
626.111	4.336	0.009	4.671	4.701	0.008	0.014
626.611	4.335	0.014	4.670	4.699	0.010	0.015
627.111	4.335	0.010	4.669	4.698	0.009	0.013
627.611	4.333	0.010	4.668	4.698	0.010	0.016
628.111	4.331	0.011	4.666	4.697	0.008	0.013
628.611	4.330	0.009	4.665	4.695	0.010	0.014
629.111	4.329	0.012	4.664	4.695	0.010	0.012
629.611	4.328	0.009	4.663	4.694	0.009	0.011
630.111	4.327	0.011	4.662	4.694	0.011	0.012
630.611	4.326	0.009	4.661	4.694	0.009	0.011
631.111	4.325	0.009	4.660	4.694	0.009	0.009
631.611	4.324	0.006	4.659	4.693	0.008	0.008
632.111	4.321	0.005	4.658	4.691	0.008	0.012
632.611	4.321	0.009	4.656	4.690	0.008	0.009
633.111	4.320	0.002	4.656	4.690	0.008	0.009
633.611	4.317	0.005	4.654	4.689	0.006	0.009
634.111	4.315	0.004	4.652	4.685	0.006	0.008
634.611	4.314	0.006	4.651	4.684	0.008	0.010
635.111	4.312	0.002	4.650	4.685	0.006	0.008
635.611	4.310	0.005	4.648	4.682	0.006	0.009
636.111	4.308	0.003	4.647	4.681	0.006	0.007

636.611	4.307	0.005	4.646	4.679	0.006	0.008
637.111	4.306	0.004	4.645	4.679	0.007	0.005
637.611	4.304	0.003	4.644	4.678	0.005	0.004
638.111	4.302	0.006	4.643	4.677	0.006	0.008
638.611	4.302	0.005	4.642	4.677	0.005	0.002
639.111	4.300	0.005	4.641	4.676	0.005	0.004
639.611	4.299	0.004	4.639	4.675	0.004	0.004
640.111	4.297	0.005	4.637	4.671	0.003	0.005
640.611	4.297	0.006	4.636	4.671	0.005	0.002
641.111	4.296	0.003	4.635	4.670	0.001	0.004
641.611	4.294	0.004	4.633	4.666	0.003	0.003
642.111	4.292	0.001	4.631	4.664	0.003	0.004
642.611	4.290	0.004	4.630	4.663	0.003	0.003
643.111	4.289	0.004	4.629	4.661	0.001	0.003
643.611	4.289	0.006	4.627	4.659	0.003	0.005
644.111	4.288	0.003	4.625	4.657	0.002	0.004
644.611	4.286	0.003	4.624	4.655	0.003	0.004
645.111	4.285	0.002	4.623	4.654	0.002	0.004
645.611	4.283	0.003	4.621	4.652	0.002	0.004
646.111	4.281	0.004	4.620	4.650	0.003	0.005
646.611	4.281	0.004	4.619	4.650	0.003	0.002
647.111	4.279	0.003	4.618	4.649	0.003	0.004
647.611	4.278	0.006	4.616	4.647	0.003	0.001
648.111	4.278	0.006	4.615	4.646	0.003	0.004
648.611	4.278	0.003	4.614	4.646	0.004	0.003
649.111	4.276	0.004	4.613	4.645	0.002	0.005
649.611	4.275	0.002	4.612	4.643	0.003	0.002
650.111	4.273	0.002	4.610	4.640	0.001	0.003
650.611	4.272	0.003	4.608	4.638	0.003	0.002
651.111	4.270	0.000	4.607	4.637	0.002	0.002
651.611	4.269	0.005	4.607	4.637	0.004	0.004
652.111	4.268	0.001	4.606	4.636	0.002	0.003
652.611	4.266	0.002	4.604	4.634	0.002	0.003
653.111	4.265	0.001	4.603	4.632	0.001	0.005
653.611	4.264	0.001	4.601	4.630	0.002	0.005
654.111	4.262	0.000	4.600	4.629	0.003	0.002
654.611	4.260	0.001	4.599	4.628	0.002	0.003
655.111	4.259	0.001	4.597	4.626	0.002	0.002
655.611	4.258	0.002	4.596	4.625	0.004	0.002
656.111	4.256	0.000	4.596	4.626	0.004	0.002
656.611	4.254	0.001	4.595	4.625	0.002	0.000
657.111	4.252	0.002	4.594	4.624	0.002	0.004

657.611	4.251	0.003	4.593	4.623	0.001	0.000
658.111	4.250	0.004	4.591	4.621	0.001	0.002
658.611	4.249	0.003	4.590	4.620	0.002	0.001
659.111	4.248	0.004	4.589	4.617	0.000	0.001
659.611	4.247	0.004	4.588	4.616	0.003	0.000
660.111	4.246	0.006	4.587	4.616	0.000	0.001
660.611	4.246	0.006	4.585	4.613	0.001	0.001
661.111	4.245	0.003	4.584	4.612	0.001	0.002
661.611	4.242	0.005	4.583	4.610	0.001	0.000
662.111	4.242	0.008	4.581	4.609	0.000	0.001
662.611	4.242	0.005	4.580	4.606	0.001	0.002
663.111	4.240	0.006	4.578	4.605	0.001	0.002
663.611	4.240	0.007	4.577	4.604	0.001	0.004
664.111	4.239	0.005	4.576	4.602	0.000	0.003
664.611	4.237	0.006	4.574	4.599	0.001	0.004
665.111	4.236	0.006	4.573	4.597	0.001	0.003
665.611	4.236	0.008	4.572	4.596	0.002	0.005
666.111	4.235	0.005	4.571	4.595	0.003	0.005
666.611	4.234	0.007	4.570	4.594	0.002	0.003
667.111	4.233	0.003	4.569	4.593	0.003	0.004
667.611	4.230	0.006	4.567	4.592	0.002	0.006
668.111	4.230	0.006	4.566	4.591	0.004	0.004
668.611	4.229	0.008	4.566	4.591	0.004	0.005
669.111	4.228	0.005	4.565	4.590	0.002	0.005
669.611	4.226	0.006	4.563	4.587	0.003	0.004
670.111	4.225	0.007	4.563	4.588	0.005	0.005
670.611	4.225	0.010	4.562	4.587	0.003	0.005
671.111	4.224	0.008	4.561	4.585	0.003	0.007
671.611	4.224	0.013	4.560	4.585	0.004	0.004
672.111	4.225	0.009	4.559	4.584	0.003	0.006
672.611	4.224	0.011	4.558	4.582	0.003	0.002
673.111	4.225	0.009	4.557	4.581	0.004	0.005
673.611	4.223	0.006	4.556	4.582	0.005	0.005
674.111	4.222	0.008	4.556	4.581	0.003	0.006
674.611	4.221	0.005	4.555	4.580	0.004	0.004
675.111	4.219	0.008	4.553	4.578	0.002	0.005
675.611	4.220	0.008	4.551	4.575	0.004	0.006
676.111	4.219	0.005	4.551	4.575	0.004	0.008
676.611	4.216	0.002	4.550	4.574	0.005	0.006
677.111	4.214	0.007	4.549	4.573	0.003	0.010
677.611	4.213	0.004	4.547	4.571	0.004	0.007
678.111	4.211	0.004	4.546	4.569	0.004	0.008

678.611	4.209	0.006	4.546	4.569	0.006	0.008
679.111	4.209	0.009	4.545	4.569	0.005	0.005
679.611	4.208	0.007	4.545	4.569	0.008	0.006
680.111	4.207	0.010	4.545	4.570	0.005	0.004
680.611	4.208	0.009	4.544	4.570	0.006	0.006
681.111	4.207	0.006	4.544	4.571	0.006	0.006
681.611	4.206	0.008	4.543	4.570	0.004	0.004
682.111	4.204	0.005	4.542	4.568	0.005	0.002
682.611	4.202	0.009	4.540	4.567	0.003	0.005
683.111	4.202	0.007	4.539	4.565	0.005	0.003
683.611	4.201	0.008	4.539	4.566	0.005	0.003
684.111	4.198	0.007	4.538	4.565	0.003	0.005
684.611	4.198	0.012	4.536	4.561	0.001	0.008
685.111	4.198	0.010	4.535	4.559	0.004	0.005
685.611	4.197	0.012	4.534	4.559	0.002	0.008
686.111	4.197	0.013	4.532	4.555	0.002	0.007
686.611	4.197	0.011	4.530	4.553	0.004	0.005
687.111	4.196	0.013	4.530	4.553	0.006	0.006
687.611	4.197	0.014	4.529	4.553	0.004	0.004
688.111	4.198	0.013	4.529	4.552	0.006	0.007
688.611	4.197	0.009	4.529	4.554	0.005	0.006
689.111	4.194	0.007	4.528	4.552	0.004	0.006
689.611	4.192	0.010	4.526	4.551	0.005	0.006
690.111	4.191	0.007	4.525	4.549	0.003	0.009
690.611	4.189	0.012	4.524	4.547	0.005	0.008
691.111	4.189	0.013	4.523	4.546	0.005	0.009
691.611	4.189	0.012	4.522	4.545	0.005	0.010
692.111	4.188	0.013	4.520	4.542	0.004	0.009
692.611	4.188	0.013	4.519	4.542	0.007	0.010
693.111	4.188	0.014	4.519	4.542	0.006	0.011
693.611	4.187	0.012	4.518	4.541	0.007	0.010
694.111	4.186	0.014	4.518	4.542	0.008	0.007
694.611	4.186	0.014	4.518	4.542	0.007	0.006
695.111	4.186	0.012	4.517	4.541	0.008	0.008
695.611	4.184	0.014	4.517	4.542	0.008	0.006
696.111	4.184	0.015	4.517	4.544	0.008	0.009
696.611	4.184	0.014	4.517	4.543	0.005	0.010
697.111	4.184	0.015	4.514	4.540	0.004	0.010
697.611	4.183	0.014	4.513	4.538	0.006	0.010
698.111	4.183	0.016	4.512	4.535	0.005	0.010
698.611	4.184	0.018	4.510	4.533	0.007	0.011
699.111	4.185	0.013	4.510	4.534	0.008	0.009

699.611	4.184	0.016	4.510	4.534	0.007	0.011
700.111	4.185	0.014	4.509	4.533	0.008	0.011
700.611	4.184	0.010	4.508	4.533	0.008	0.010
701.111	4.183	0.015	4.508	4.533	0.008	0.011
701.611	4.184	0.009	4.507	4.533	0.007	0.012
702.111	4.182	0.008	4.506	4.531	0.008	0.011
702.611	4.180	0.008	4.506	4.532	0.009	0.012
703.111	4.179	0.009	4.505	4.532	0.007	0.011
703.611	4.179	0.011	4.504	4.530	0.008	0.013
704.111	4.179	0.006	4.504	4.530	0.009	0.014
704.611	4.176	0.006	4.504	4.530	0.008	0.010
705.111	4.177	0.010	4.503	4.530	0.009	0.012
705.611	4.177	0.003	4.502	4.529	0.008	0.011
706.111	4.174	0.003	4.502	4.529	0.010	0.008
706.611	4.172	0.002	4.502	4.532	0.011	0.012
707.111	4.169	0.001	4.502	4.532	0.008	0.007
707.611	4.166	0.003	4.502	4.532	0.010	0.006
708.111	4.166	0.006	4.502	4.534	0.008	0.006
708.611	4.164	0.003	4.501	4.532	0.006	0.007
709.111	4.161	0.007	4.500	4.532	0.009	0.008
709.611	4.163	0.012	4.500	4.533	0.005	0.005
710.111	4.164	0.007	4.499	4.530	0.005	0.004
710.611	4.162	0.006	4.497	4.528	0.005	0.008
711.111	4.160	0.007	4.496	4.526	0.006	0.003
711.611	4.159	0.008	4.496	4.528	0.006	0.002
712.111	4.159	0.012	4.496	4.527	0.004	0.001
712.611	4.159	0.010	4.494	4.524	0.003	0.001
713.111	4.158	0.009	4.494	4.525	0.006	0.002
713.611	4.158	0.013	4.494	4.525	0.002	0.004
714.111	4.159	0.011	4.492	4.522	0.002	0.002
714.611	4.159	0.011	4.490	4.519	0.001	0.005
715.111	4.158	0.009	4.488	4.515	0.001	0.009
715.611	4.158	0.011	4.486	4.512	0.002	0.005
716.111	4.157	0.006	4.486	4.511	0.003	0.004
716.611	4.156	0.010	4.484	4.509	0.002	0.006
717.111	4.155	0.005	4.482	4.506	0.004	0.006
717.611	4.153	0.009	4.483	4.508	0.007	0.009
718.111	4.152	0.008	4.483	4.510	0.004	0.008
718.611	4.151	0.010	4.482	4.507	0.003	0.007
719.111	4.151	0.010	4.480	4.505	0.005	0.010
719.611	4.151	0.009	4.479	4.503	0.005	0.008
720.111	4.151	0.010	4.479	4.504	0.007	0.008

720.611	4.150	0.006	4.479	4.505	0.006	0.007
721.111	4.148	0.008	4.478	4.503	0.005	0.008
721.611	4.146	0.006	4.478	4.503	0.008	0.004
722.111	4.144	0.009	4.478	4.505	0.007	0.008
722.611	4.145	0.012	4.478	4.505	0.007	0.004
723.111	4.145	0.010	4.477	4.505	0.006	0.007
723.611	4.145	0.013	4.477	4.505	0.007	0.006
724.111	4.146	0.008	4.476	4.504	0.004	0.007
724.611	4.144	0.009	4.475	4.502	0.006	0.007
725.111	4.143	0.010	4.474	4.501	0.003	0.007
725.611	4.142	0.010	4.473	4.499	0.005	0.007
726.111	4.142	0.010	4.472	4.498	0.005	0.004
726.611	4.141	0.008	4.471	4.497	0.006	0.006
727.111	4.140	0.011	4.471	4.498	0.006	0.005
727.611	4.141	0.010	4.470	4.497	0.005	0.007
728.111	4.139	0.006	4.470	4.497	0.006	0.009
728.611	4.138	0.012	4.469	4.496	0.003	0.008
729.111	4.138	0.008	4.468	4.494	0.005	0.010
729.611	4.136	0.010	4.466	4.492	0.004	0.006
730.111	4.137	0.010	4.465	4.489	0.005	0.007
730.611	4.135	0.006	4.465	4.490	0.007	0.007
731.111	4.133	0.008	4.465	4.490	0.006	0.007
731.611	4.131	0.008	4.465	4.492	0.008	0.007
732.111	4.131	0.014	4.465	4.492	0.005	0.006
732.611	4.131	0.008	4.463	4.490	0.005	0.008
733.111	4.129	0.011	4.463	4.489	0.006	0.007
733.611	4.130	0.014	4.462	4.488	0.006	0.004
734.111	4.129	0.010	4.462	4.488	0.006	0.009
734.611	4.129	0.015	4.461	4.487	0.005	0.006
735.111	4.130	0.013	4.460	4.486	0.006	0.007
735.611	4.130	0.011	4.460	4.487	0.006	0.008
736.111	4.128	0.010	4.458	4.484	0.003	0.004
736.611	4.126	0.012	4.458	4.483	0.007	0.006
737.111	4.125	0.013	4.457	4.483	0.005	0.006
737.611	4.126	0.016	4.456	4.482	0.006	0.010
738.111	4.127	0.012	4.456	4.483	0.006	0.006
738.611	4.125	0.011	4.455	4.481	0.004	0.008
739.111	4.124	0.012	4.454	4.478	0.005	0.010
739.611	4.123	0.013	4.452	4.475	0.005	0.008
740.111	4.122	0.013	4.452	4.476	0.008	0.012
740.611	4.120	0.013	4.452	4.476	0.005	0.010
741.111	4.120	0.018	4.450	4.473	0.006	0.008

741.611	4.120	0.014	4.450	4.474	0.008	0.007
742.111	4.119	0.018	4.450	4.474	0.006	0.009
742.611	4.120	0.018	4.449	4.474	0.009	0.010
743.111	4.121	0.020	4.450	4.476	0.008	0.012
743.611	4.121	0.014	4.449	4.475	0.007	0.009
744.111	4.120	0.017	4.448	4.473	0.006	0.008
744.611	4.120	0.018	4.447	4.471	0.007	0.009
745.111	4.118	0.015	4.446	4.470	0.008	0.009
745.611	4.118	0.021	4.446	4.472	0.009	0.009
746.111	4.118	0.017	4.446	4.472	0.007	0.009
746.611	4.117	0.020	4.445	4.470	0.007	0.013
747.111	4.118	0.021	4.444	4.469	0.007	0.010
747.611	4.119	0.022	4.443	4.468	0.008	0.013
748.111	4.121	0.022	4.442	4.467	0.008	0.013
748.611	4.121	0.021	4.441	4.464	0.008	0.015
749.111	4.123	0.021	4.441	4.465	0.011	0.011
749.611	4.123	0.016	4.441	4.465	0.008	0.013
750.111	4.122	0.017	4.440	4.464	0.011	0.013
750.611	4.121	0.011	4.440	4.465	0.011	0.011
751.111	4.118	0.013	4.441	4.468	0.012	0.015
751.611	4.117	0.016	4.441	4.467	0.009	0.013
752.111	4.118	0.015	4.439	4.465	0.010	0.015
752.611	4.116	0.013	4.439	4.466	0.011	0.016
753.111	4.116	0.016	4.438	4.464	0.009	0.016
753.611	4.116	0.014	4.438	4.463	0.013	0.016
754.111	4.114	0.013	4.438	4.464	0.011	0.015
754.611	4.113	0.016	4.437	4.463	0.012	0.016
755.111	4.113	0.014	4.437	4.465	0.013	0.012
755.611	4.112	0.016	4.438	4.467	0.013	0.012
756.111	4.113	0.016	4.438	4.468	0.013	0.008
756.611	4.114	0.018	4.438	4.470	0.013	0.010
757.111	4.113	0.012	4.439	4.472	0.013	0.012
757.611	4.112	0.018	4.439	4.473	0.010	0.011
758.111	4.114	0.016	4.438	4.472	0.010	0.010
758.611	4.112	0.010	4.437	4.470	0.007	0.012
759.111	4.111	0.018	4.435	4.466	0.008	0.010
759.611	4.111	0.014	4.435	4.465	0.010	0.009
760.111	4.112	0.018	4.435	4.466	0.009	0.012
760.611	4.113	0.015	4.434	4.464	0.008	0.010
761.111	4.113	0.012	4.433	4.464	0.010	0.012
761.611	4.109	0.007	4.433	4.464	0.008	0.012
762.111	4.108	0.016	4.432	4.462	0.008	0.013

762.611	4.109	0.011	4.431	4.461	0.010	0.009
763.111	4.109	0.012	4.431	4.461	0.009	0.013
763.611	4.107	0.008	4.430	4.460	0.010	0.011
764.111	4.104	0.009	4.430	4.461	0.010	0.008
764.611	4.103	0.012	4.430	4.463	0.011	0.013
765.111	4.103	0.014	4.430	4.462	0.008	0.010
765.611	4.103	0.013	4.429	4.461	0.011	0.013
766.111	4.103	0.014	4.430	4.464	0.010	0.011
766.611	4.103	0.015	4.428	4.461	0.006	0.008
767.111	4.102	0.013	4.427	4.459	0.011	0.005
767.611	4.104	0.020	4.428	4.460	0.009	0.011
768.111	4.107	0.016	4.428	4.461	0.011	0.008
768.611	4.106	0.010	4.428	4.464	0.009	0.009
769.111	4.103	0.012	4.428	4.463	0.007	0.006
769.611	4.103	0.015	4.426	4.459	0.005	0.007
770.111	4.103	0.011	4.425	4.458	0.010	0.009
770.611	4.103	0.015	4.425	4.459	0.007	0.010
771.111	4.104	0.013	4.425	4.458	0.007	0.009
771.611	4.104	0.012	4.424	4.457	0.005	0.010
772.111	4.103	0.012	4.422	4.453	0.006	0.011
772.611	4.103	0.012	4.421	4.451	0.008	0.009
773.111	4.105	0.011	4.421	4.452	0.009	0.015
773.611	4.102	0.003	4.420	4.452	0.008	0.011
774.111	4.100	0.010	4.420	4.451	0.009	0.007
774.611	4.100	0.006	4.420	4.452	0.009	0.008
775.111	4.097	0.004	4.419	4.451	0.008	0.011
775.611	4.096	0.008	4.420	4.453	0.012	0.008
776.111	4.094	0.006	4.422	4.458	0.010	0.011
776.611	4.093	0.010	4.421	4.456	0.006	0.009
777.111	4.095	0.012	4.419	4.453	0.007	0.009
777.611	4.094	0.008	4.419	4.453	0.009	0.008
778.111	4.092	0.010	4.418	4.453	0.007	0.009
778.611	4.093	0.013	4.418	4.453	0.009	0.008
779.111	4.095	0.013	4.418	4.455	0.008	0.002
779.611	4.095	0.009	4.418	4.455	0.008	0.007
780.111	4.094	0.008	4.418	4.454	0.007	0.005
780.611	4.092	0.009	4.418	4.455	0.007	0.003
781.111	4.093	0.012	4.418	4.457	0.007	0.006
781.611	4.093	0.009	4.417	4.453	0.002	0.004
782.111	4.093	0.012	4.415	4.451	0.006	0.007
782.611	4.094	0.008	4.415	4.452	0.004	0.008
783.111	4.094	0.011	4.413	4.448	0.002	0.005

783.611	4.096	0.007	4.412	4.445	0.005	0.007
784.111	4.093	0.001	4.411	4.444	0.004	0.009
784.611	4.091	0.007	4.410	4.443	0.006	0.009
785.111	4.090	0.004	4.411	4.445	0.007	0.006
785.611	4.089	0.005	4.410	4.444	0.005	0.006
786.111	4.087	0.001	4.409	4.442	0.006	0.006
786.611	4.086	0.008	4.409	4.443	0.008	0.009
787.111	4.087	0.006	4.410	4.446	0.008	0.006
787.611	4.087	0.004	4.410	4.447	0.005	0.009
788.111	4.085	0.005	4.409	4.445	0.005	0.006
788.611	4.086	0.005	4.408	4.443	0.006	0.008
789.111	4.085	0.002	4.408	4.444	0.008	0.005
789.611	4.082	0.001	4.408	4.445	0.005	0.001
790.111	4.080	0.004	4.408	4.445	0.008	0.005
790.611	4.078	0.002	4.409	4.446	0.005	0.003
791.111	4.077	0.010	4.409	4.447	0.007	0.003
791.611	4.078	0.009	4.409	4.449	0.004	0.001
792.111	4.080	0.014	4.407	4.445	0.001	0.006
792.611	4.083	0.009	4.406	4.443	0.004	0.005
793.111	4.080	0.004	4.406	4.443	0.002	0.003
793.611	4.077	0.007	4.405	4.442	0.003	0.004
794.111	4.076	0.011	4.404	4.439	0.001	0.004
794.611	4.079	0.016	4.403	4.438	0.005	0.001
795.111	4.082	0.012	4.404	4.440	0.004	0.001
795.611	4.084	0.014	4.403	4.440	0.002	0.003
796.111	4.085	0.006	4.402	4.439	0.003	0.001
796.611	4.083	0.007	4.403	4.440	0.003	0.007
797.111	4.083	0.004	4.402	4.439	0.001	0.006
797.611	4.079	0.002	4.400	4.436	0.001	0.006
798.111	4.078	0.009	4.399	4.434	0.003	0.006
798.611	4.080	0.008	4.398	4.433	0.001	0.006
799.111	4.081	0.006	4.399	4.433	0.006	0.006
799.611	4.082	0.007	4.401	4.440	0.006	0.006

REFERENCES

(1) Li, L.-S.; Alivisatos, A. P. Origin and Scaling of the Permanent Dipole Moment in CdSe Nanorods. *Phys. Rev. Lett.* **2003**, *90* (9),

(2) Yamamoto, J.; Oura, M.; Yamashita, T.; Miki, S.; Jin, T.; Haraguchi, T.; Hiraoka, Y.; Terai, H.; Kinjo, M. Rotational diffusion measurements using polarization-dependent fluorescence

correlation spectroscopy based on superconducting nanowire single-photon detector. *Opt. Express* **2015**, *23* (25), 32633-32642.

(3) Lakowicz, J. R. *Principles of Fluorescence Spectroscopy*. 3rd ed.; Springer: Boston, MA, 2006; pp 291-319.

(4) Tice, D. B.; Weinberg, D. J.; Mathew, N.; Chang, R. P. H.; Weiss, E. A. Measurement of Wavelength-Dependent Polarization Character in the Absorption Anisotropies of Ensembles of CdSe Nanorods. *J. Phys. Chem. C* **2013**, *117* (25), 13289-13296.

(5) Sitt, A.; Salant, A.; Menagen, G.; Banin, U. Highly Emissive Nano Rod-in-Rod Heterostructures with Strong Linear Polarization. *Nano Lett.* **2011**, *11* (5), 2054-2060.

(6) Diroll, B. T.; Dadosh, T.; Koschitzky, A.; Goldman, Y. E.; Murray, C. B. Interpreting the Energy-Dependent Anisotropy of Colloidal Nanorods Using Ensemble and Single-Particle Spectroscopy. *J. Phys. Chem. C* **2013**, *117* (45), 23928-23937.

(7) Ratnaweera, R. J.; Rodríguez Ortiz, F. A.; Gripp, N. J.; Sheldon, M. T. Quantifying Order during Field-Driven Alignment of Colloidal Semiconductor Nanorods. *ACS Nano* **2022**, *16* (3), 3834-3842.

(8) Jones, R. C. A Generalization of the Dielectric Ellipsoid Problem. *Physical Review* **1945**, *68* (3-4), 93-96.

(9) Sihvola, A. *Electromagnetic Mixing Formulas And Applications*. 1st ed.; The Institution of Engineering and Technology: London, United Kingdom, 1999; pp 63-70.

(10) Moreels, I.; Allan, G.; De Geyter, B.; Wirtz, L.; Delerue, C.; Hens, Z. Dielectric function of colloidal lead chalcogenide quantum dots obtained by a Kramers-Krönig analysis of the absorbance spectrum. *Phys. Rev. B* **2010**, *81* (23), 235319.

(11) Dement, D. B.; Puri, M.; Ferry, V. E. Determining the Complex Refractive Index of Neat CdSe/CdS Quantum Dot Films. *J. Phys. Chem. C* **2018**, *122* (37), 21557-21568.

(12) Mannino, G.; Deretzis, I.; Smecca, E.; Giannazzo, F.; Valastro, S.; Fisicaro, G.; La Magna, A.; Ceratti, D.; Alberti, A. $CsPbBr_3$, $MAPbBr_3$, and $FAPbBr_3$ Bromide Perovskite Single Crystals: Interband Critical Points under Dry N_2 and Optical Degradation under Humid Air. *J. Phys. Chem. C* **2021**, *125* (9), 4938-4945.
